# Stimuli‐Responsive Sponge for Imaging and Measuring Weak Compression Stresses

**DOI:** 10.1002/advs.202206097

**Published:** 2022-12-11

**Authors:** Nahoko Ono, Ryo Seishima, Koji Okabayashi, Hiroaki Imai, Syuji Fujii, Yuya Oaki

**Affiliations:** ^1^ Department of Applied Chemistry Faculty of Science and Technology Keio University 3‐14‐1 Hiyoshi Kohoku‐ku Yokohama 223–8522 Japan; ^2^ Department of Surgery School of Medicine Keio University 35 Shinanomachi, Shinjuku‐ku Tokyo 160–8582 Japan; ^3^ Department of Applied Chemistry Faculty of Engineering Osaka Institute of Technology 5‐16‐1 Omiya Asahi‐ku Osaka 535–8585 Japan

**Keywords:** layered materials, polydiacetylene, sponge devices, stimuli responsiveness, stress imaging

## Abstract

Imaging and measuring compression stresses secure a safe and healthy life. Compression stresses in kPa range are not easily detected by conventional mechanoresponsive materials because microscopic molecular motion of the chromophores is not induced by such weak stresses. Moreover, imaging of the stress distribution is not achieved so far. The present study shows a sponge device combining two stimuli‐responsive materials, a capsule releasing interior liquid and color‐changing polymer in responses to compression stress and chemical stimulus, respectively. The stimuli‐responsive capsule is dispersed on a melamine sponge comprised of the fibers with coating the layered polydiacetylene (PDA). The application of weak compression stresses induces collapse of the capsules, outflow of the interior liquid, and subsequent irreversible color change of PDA. The cascading response in the sponge device colorimetrically enables imaging of the distribution and measuring the strength of the compression stresses in kPa range. Furthermore, the device demonstrates imaging and measuring unknown weak compression stresses applied by the irregular‐shaped objects. A couple of clinical issues in surgical operation of intestine are studied using the stress‐imaging sponge device. The device and its design strategy can be applied to stress imaging in a variety of fields.

## Graphical abstract

Imaging invisible force distribution is a significant challenge in chemistry and materials science. The present work shows stimuli‐responsive sponge device for imaging and measuring weak compression stresses in kPa range and mm scale. The device visualizes and quantifies the local compression stress and its distribution applied by irregularly shaped objects, such as plastic ornaments and surgical devices.

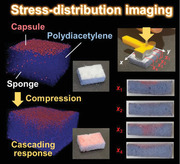

## 1 Introduction

**Figure 1:**
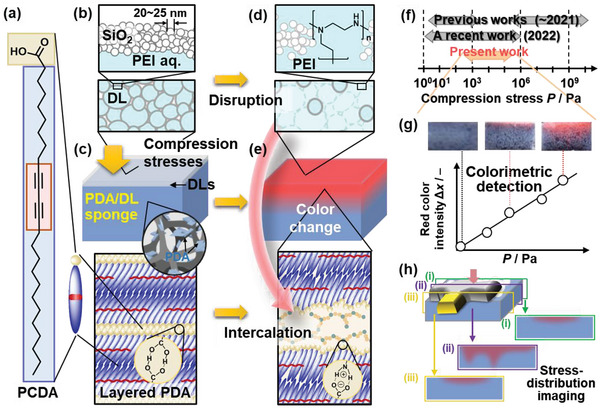
Overview of the PDA/DL sponge device and its application. a) PCDA monomer. b) DL consisting of interior PEI solution and shell SiO_2_ particles dispersed on the surface of the sponge. c) PDA/DL sponge device with coating of the layered PDA in the fibrous network. d) Disruption of DLs and subsequent outflow of the interior liquid containing PEI with the application of compression stress. e) Color change of PDA with the intercalation of PEI in the interlayer space. f) Sensing range of compression stresses in the previous works,^[^ ^21^, ^22^, ^23^, ^24^, ^25^, ^26^, ^27^, ^28^, ^29^, ^30^, ^31^, ^32^, ^33^, ^34^, ^35^, ^36^, ^37^, ^38^, ^39^, ^40^ ^]^ a recent work (gray),^[^ ^41^ ^]^ and present work (orange). g) Representative cross‐sectional photographs of the sponge device and relationship between *P* and ∆*x* for the colorimetric detection. h) Imaging the compression–stress distribution in the different cross sections.

Compression stresses are found in a variety of strength ranges and length scales.^[^ ^1^, ^2^, ^3^, ^4^ ^]^ For example, experienced physicians, craftsmen, and artists handle tools and objects with their fine‐tuned forces. If strength and its distribution of the compression stresses are visualized and measured, the professional motions can be elucidated and inherited to the next generation. Strain gauge and piezoelectric device are conventional force sensors.^[^ ^5^, ^6^ ^]^ However, these devices have limitations to the strength and length scales for imaging and measuring. Although electronic skin devices have extensively been studied in recent years,^[^ ^7^, ^8^ ^]^ imaging the strength and its distribution still remain challenges. In the present work, a sponge device integrating two stimuli‐responsive materials was designed for imaging and measuring weak compression stresses in kPa range and mm scale (**Figure** **1**).

Mechanoresponsive color‐change materials have been studied in recent years.^[^ ^9^, ^10^, ^11^, ^12^, ^13^, ^14^, ^15^, ^16^, ^17^, ^18^ ^]^ The designed chromophores and their organized materials show the color changes with the application of mechanical stresses. For example, aggregation‐induced emission is used for detection of compression stresses.^[^ ^19^, ^20^ ^]^ However, the detection range of compression stresses was limited to 10^0^–10^3^ Pa and 10^5^–10^10^ Pa in 2021 (Figure 1f; Figure S1, Supporting Information).^[^ ^21^, ^22^, ^23^, ^24^, ^25^, ^26^, ^27^, ^28^, ^29^, ^30^, ^31^, ^32^, ^33^, ^34^, ^35^, ^36^, ^37^, ^38^, ^39^, ^40^ ^]^ The compression stresses in the range of 10^3^–10^5^ Pa were not detected because the color changes with molecular motion of chromophores are not induced by such weak mechanical stresses. A recent report shows that a polymer gel containing the flapping force probe enables the compression stress imaging in the range of 10^0^–10^6^ Pa (Figure 1f).^[^ ^41^ ^]^ However, the detection needs excitation exhibiting fluorescence. In addition, if the sensitivity is enhanced by molecular design, the color change can be caused by thermal motion under ambient conditions without stimulation before sensing. New design strategies of materials and devices are required to achieve high sensitivity. Moreover, imaging the stress distribution needs specific design of materials and devices. Here, cascading response scheme was introduced in a commercial melamine sponge to achieve imaging and measuring of weak compression stresses (Figure 1a–c). Disruption of capsules containing interior liquid, dry liquid (DL), was induced by the application of weak compression stress (Figure 1b,d). The outflowed liquid as a chemical stress irreversibly changed the color of layered PDA in the sponge (Figure 1d,e). The color‐changed sponge device enabled visible imaging and measuring of weak compression stresses without excitation light in kPa range and mm scale, that were not achieved in previous mechanoresponsive materials (Figure 1f–h).

PDA is a stimuli‐responsive color‐change polymer applicable to sensing.^[^ ^42^, ^43^, ^44^, ^45^, ^46^, ^47^, ^48^, ^49^, ^50^ ^]^ The original blue color is changed to red with the application of external stimuli through shortening of the effective conjugation length. The stimuli responsivity of PDA is controlled by molecular design and intercalation approaches.^[^ ^51^, ^52^, ^53^, ^54^, ^55^, ^56^, ^57^, ^58^, ^59^, ^60^ ^]^ In general, PDA is synthesized by topochemical polymerization of diacetylene (DA) monomers in condensed states, such as layered structures and vesicles.^[^ ^61^ ^]^ Whereas color changes in response to thermal and chemical stresses were extensively studied, responsiveness to mechanical stress was limited in previous works.^[^ ^49^, ^50^, ^55^, ^60^, ^62^, ^63^, ^64^ ^]^ In a recent report, the strain applied to the PDA‐contained hydrogel was detected by changes in not the color but the capacitance.^[^ ^65^ ^]^ Ultrasonic stress was detected as the shear stress using self‐assembled PDA vesicles.^[^ ^66^ ^]^ Another recent report indicates that the mechanoresponsive color change of PDA is dominantly induced by shear stress applied parallel to the layered structures.^[^ ^67^ ^]^ These facts indicate that the stimuli‐responsive molecular motion of PDA is not induced by compression and tensile stresses. Therefore, new design strategies of materials and devices are required to achieve color changes in response to mechanical stresses, including compression and tensility. In our previous work, compression stress was detected by combination of DL and PDA on a paper substrate.^[^ ^40^ ^]^ However, the detection was limited to ultraweak compression stresses in the range of 10^0^–10^3^ Pa without the tunability. The visible imaging of the stress distribution and measuring in the range of 10^3^–10^5^ Pa have not been achieved without excitation light in previous works about mechanoresponsive materials (Figure 1f; Figure S1 in the Supporting Information).^[^ ^21^, ^22^, ^23^, ^24^, ^25^, ^26^, ^27^, ^28^, ^29^, ^30^, ^31^, ^32^, ^33^, ^34^, ^35^, ^36^, ^37^, ^38^, ^39^, ^40^, ^41^ ^]^The present work shows a new stimuli‐responsive sponge device with integration of PDA and DL (Figure 1). Polyethyleneimine (PEI) flow out of DLs is diffused into the sponge coated by PDA and then interacted in the interlayer space of PDA (Figure 1c,e). The intercalation induces the molecular motion of the PDA main chain, leading to the color change through shortening the effective conjugation length (Figure 1e). 3D sponge plays important roles for the tunable responsivity and stress‐distribution imaging. The applied strength is recorded by the irreversible color change in the sponge. The device is successfully applied to estimate unknown weak compression stresses in the local area and visualize the stress distribution applied by irregularly shaped objects (Figure 1f–h). The sponge device and its design strategy can be applied to imaging compression stresses in a variety of strength ranges and length scales.

## 2 Results and Discussion

### 2.1 Sponge Device Integrating PDA and DL

**Figure 2:**
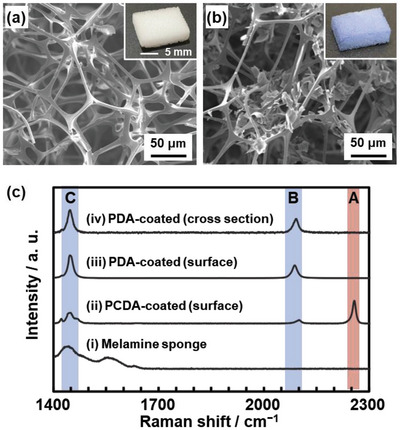
Structure of the PDA‐coated sponge. a,b) SEM images and photographs (inset) of a melamine sponge (a) and PDA‐coated sponge (b). c‐i) Raman spectra of a melamine sponge, c‐ii) PCDA‐monomer‐coated melamine sponge, c‐iii) surface, and c‐iv) cross section of the PDA‐coated melamine sponge.

A commercial melamine sponge (20 × 10 × 5 mm) with the average pore diameter of 36.7 ± 37.9 µm was immersed in acetone solution containing a DA monomer, 10,12‐pentacosadiynoic acid (PCDA) (Figures 1a and **2**a; Figure S2, Supporting Information). Amphiphilic PCDA formed the layered crystal structure, as shown in Figure 1a,c, with evaporation of the solvent. After the dip‐coating, the blue PDA‐coated melamine sponge was obtained with the irradiation of UV light (254 nm) from both the top and bottom sides (Figure 2b). As UV light partially permeated the melamine sponge, ≈5% of transmittance at 254 nm (Figure S2, Supporting Information), the polymerization of PCDA proceeded in the sponge. The detailed procedure is described in the Supporting Information. PDA sheets 4.5 ± 2.4 µm in lateral size and 120 ± 91 nm in thickness were observed on the interior fibers of the sponge by scanning electron microscopy (SEM) (Figure 2a,b). The weight of the sponge 13.7 ± 0.6 mg increased to 16.0 ± 0.5 mg after the deposition of PDA (Table S1, Supporting Information).

Raman spectra indicate the formation of PDA (Figure 2c). The PCDA‐coated melamine sponge showed the peak around 2260 cm^−1^ corresponding to C≡C bond (spectra [i,ii] with band A in Figure 2c). After the polymerization, the peaks around 2090 and 1450 cm^−1^ corresponding to the ene‐yne structure of C≡C and C=C bonds appeared with the disappearance of the monomer peak, respectively (spectra [iii,iv] with bands B and C in Figure 2c). The spectroscopic changes were observed on both the surface and cross section of the sponge. As the remaining peak corresponding to the monomer was not observed, the topochemical polymerization of PCDA was achieved in the whole of the sponge.

DL was prepared with mixing 20 wt% branched polyethyleneimine (PEI, $\bar{M}$ *_n_* = 300) aqueous solution and polydimethylsiloxane (PDMS)‐coated silica (SiO_2_) nanoparticles, according to the previous report.^[^ ^68^ ^]^ DL in the range of 125–250 µm, average diameter 148 ± 59 µm, was collected using sieves (Figure S3, Supporting Information). The extracted DL contained 92–94 wt% of the interior PEI solution (Table S2, Supporting Information). The resultant DL was stored in a plastic bottle after the preparation until the use (Figure S3, Supporting Information). DL, typically ≈10 mg, was taken from the bottle dispersed on the surface of the PDA‐coated sponge (≈15 mg) (Figure 1b,c).

### 2.2 Color Changes in Response to Weak Compression Stresses

**Figure 3:**
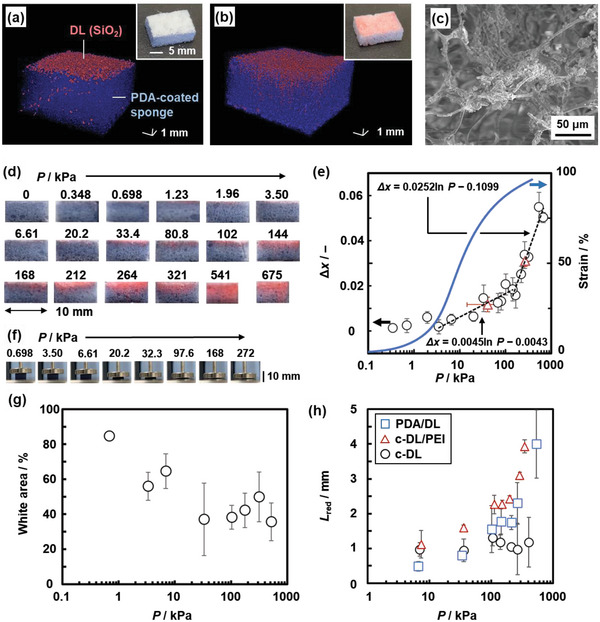
Color‐change behavior of the PDA/DL sponge. a,b) XCT stereoscopic images and photographs (inset) of the PDA/DL sponge device before (a) and after (b) the application of compression stress. c) SEM image of the inside of the sponge after the application of compression stress. d) Cross‐sectional photographs of the PDA/DL sponge device with the application of *P* = 0.348–675 kPa. e) Relationship between *P* and ∆*x* (black circles, left axis) with the colorimetric estimation of unknown compression stresses (red triangles) and stress–strain curve of the sponge device (blue, right axis) (sample size: *n* = 3). f) Photographs of the compressed sponge devices. g) Relationship between *P* and white‐color area on the surface of the sponge device originating from the remaining DL with the compression before the removal (sample size: *n* = 3). h) Relationship between *P* and *L*_red_ of the PDA/DL sponge and reference white sponge withc‐DL and c‐DL/PEI (sample size: *n* = 3).

Compression stress was applied to the 3D PDA/DL sponge device. The color change of the sponge was observed after removal of the remaining DLs (the insets of **Figure** **3**a,b). The PDA/DL sponge device showed the mechanoresponsiveness. In the initial state, the SiO_2_ particles originating from DLs were observed as the red‐color domain on the surface of the sponge by X‐ray computer tomography (XCT) (Figure 3a). After the application of the compression stress, the red‐color domain was expanded to the inside of the sponge (Figure 3b). The fragmented SiO_2_ particles were observed inside of the sponge by SEM with energy‐dispersive X‐ray (EDX) analysis (Figure 3c; Figure S4, Supporting Information). The results imply that disruption of DL induces outflow and diffusion of the interior PEI solution inside of the sponge, leading to the color change (Figure 3b,c). Intercalation of PEI in the interlayer space of the layered PDA acts as a trigger to induce the torsion of the PDA main chain, leading to the color change (Figure 1c,d). PDA in the initial state showed the absorption band around 1700 cm^−1^ corresponding to dimerized carboxy groups on Fourier‐transform infrared (FT‐IR) spectrum (Figure S4, Supporting Information). The absorption band around 1570 cm^−1^ corresponding to carboxylate group appeared after the color change with the application of compression stress. The changes in the state of the interlayer carboxy group imply the intercalation of PEI (Figure 1c,d). The X‐ray diffraction (XRD) peak corresponding to the interlayer distance was broadened and shifted to the lower‐angle region after the color change (Figure S4, Supporting Information). An increase in the interlayer distance supports the intercalation of PEI. PDA normally shows no responses to mechanical stimuli except friction force. In the present work, the cascading responses through generation of chemical stress facilitate the response to compression stress.

The PDA/DL 3D sponge device was compressed by the stresses in the range of 0–675 kPa using a tester via a probe 30 mm in diameter for 10 s (Figure 3d). The remaining DL was removed using a handy brower within 10 s (Figure S5, Supporting Information). Then, the sponge was cut and the photograph of the cross section was taken to estimate the increment of the red‐color intensity (∆*x*) in the area of 5 × 10 mm (Figure 3d). The photographs were taken at 100 s after removal of DLs. The time for the readout (*t*_r_) after cutting the device has no influence on Δ*x* (Figure S5, Supporting Information). The time for sensing, including the compression, removal of the remaining DLs, and taking photographs (readout time), was an almost constant for the reproducibility (Scheme S1, Supporting Information). The ∆*x* values were increased with increasing the applied compression stress (*P*) (Figure 3d,e). The photographs indicate that *P* in the range of 1.23–675 kPa was visualized by the red color (Figure 3d). The specific relationship between *P* and ∆*x* was observed in the range of 3.50–675 kPa (Figure 3e; Figure S6 and Table S3, Supporting Information). The relationship as the standard curve can be used for colorimetric estimation of unknown compression stresses. Simulated unknown compression stresses, namely 50.1 ± 2.80 and 209 ± 8.70 kPa, were applied to the device. The colorimetrically estimated values were 40.9 ± 26.5 and 269 ± 26.2 kPa from ∆*x* = 0.012 ± 0.003 and 0.031± 0.003, respectively (the red triangles in Figure 3e). Whereas the weak compression stresses in the range of 3.50–200 kPa were not detected without excitation in previous works (Figure S1, Supporting Information), the PDA/DL device enables imaging and measuring the weak compression stresses. The standard curve can be applied to estimate unknown *P*. Moreover, the sensitivity was tuned by changes in the loaded amount of DL and softness of the sponge (Figures S7 and S8 and Tables S4 and S5, Supporting Information). When the applied duration of the compression (*t*_c_) was changed in the range of 1 to 300 s, the Δ*x* values increased in the range of *t*_c_ = 1 to 100 s and reached the constant value in the range of *t*_c_ = 100 to 300 s (Figure S9, Supporting Information). The results indicate that *t*_c_ can be estimated from Δ*x* under certain *P*.

In the present work, DL was set on the top surface of the PDA‐coated sponge (the inset of Figure 3a) because the average pore size of the sponge and diameter of DL were 36.7 ± 37.9 µm and 148 ± 59 µm, respectively. When the size‐selected DLs were embedded in the inside of the PDA‐coated sponge, the colorimetric response was not observed to the applied compression stresses (Figure S10, Supporting Information). The disruption of DLs leading to the color changes is not achieved in response to the applied stress. Therefore, sensing the compression stress requires the sponge with the pore size smaller than the droplet size.

### 2.3 Colorimetric Response to Weak Compression Stresses

Disruption of DL and diffusion of PEI in the sponge enable the colorimetric response to *P* (Figure 3d,e). The strained sponge promotes the diffusion of PEI (Figure 3f). In the present work, DL was set on the surface of the sponge device (Figure 3a). Whereas the disrupted DL was infiltrated in the sponge with the application of compression stress, the remaining pristine DL was removed from the device before measuring ∆*x* on the cross section. The ratio of the remaining DL was estimated from the white‐color area on the surface before the removal from the sponge. The white‐color area corresponding to the number of the remaining DL without disruption decreased with increasing *P* in the range of 1–50 kPa and then became constant (Figure 3g). According to these results, the number of the disrupt DL increases with increasing *P* in the range of 1–50 kPa.

The further increase in *P* promotes the diffusion of PEI in the inside of the sponge with the large strain. The disrupt DLs were observed near the surface layer of the sponge regardless of *P* (Figure 3b; Figure S11, Supporting Information). Nevertheless, the color changes depending on *P* were visible in the deeper area of the sponge (Figure 3d). These results imply that the diffusion depth of PEI into the sponge increases with increasing *P*. As PEI ($\bar{M}$ *_n_* = 300) used in the present work is in a liquid state, the diffusion into sponge is continued after evaporation of water. The diffusion behavior of PEI was studied by combination of the pristine white sponge and colored DL (c‐DL) (Figure 3h; Figure S12 and Table S6, Supporting Information). Two types of c‐DLs were prepared with purified water and PEI solution containing a red dye (rhodamine B). These c‐DLs were set on the white sponge without PDA coating. The depth of the red‐colored area from the surface (*L*_Red_) was measured with the application of compression stresses. c‐DL containing PEI (c‐DL/PEI) showed the larger ∆*x* and *L*_Red_ in the sponge compared with c‐DL without PEI (circles and triangles in Figure 3h; Figure S13 and Table S6, Supporting Information). The PDA/DL device showed the similar *L*_Red_ (triangles and squares in Figure 3h). The diffusion of PEI enhances the color change in the sponge. The photographs and stress–strain curves of the sponge device indicate that the diffusion of PEI is promoted in the strained state (Figure 3e,f). Therefore, the colorimetric response originates from both the disruption ratio of DLs and diffusion depth of PEI depending on *P* (Figure 3g,h). The responsivity of the reference devices using c‐DLs was quite low compared with that of the PDA/DL device (Figure S13 and Table S6, Supporting Information). The cascading responses of PDA/DL are required for sensing weak compression stresses. The cascading response with combination of PDA and DL was demonstrated in our previous work.^[^ ^40^ ^]^ In the present work, the sensing properties were tuned by the diffusion control of the interior liquid using 3D sponge. The sponge as an elastic matrix had effects on the sensing range. Whereas the compression stresses in the range of 3.9 Pa–4.9 kPa were detected by PDA/DL on a paper substrate,^[^ ^40^ ^]^ the sponge device visualized and quantified the stresses in the range of 3.50–674 kPa in the present work. The elasticity of the sponge tuned the diffusion behavior of the interior liquid, leading to changes in the sensitivity. Moreover, the stress distribution mapping was achieved by the PDA/DL sponge device. In this manner, our design strategy using a sponge enables the sensitivity tuning and distribution mapping, that are not achieved by conventional mechanoresponsive materials based on molecular design.

### 2.4 Measurement of Unknown Compression Stresses

**Figure 4:**
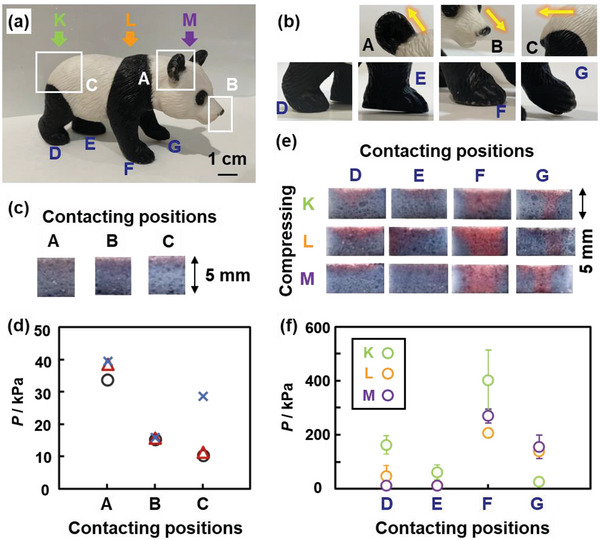
Colorimetric estimation of unknown compression stresses. a) Photograph of a plastic ornament of giant panda. b) Magnified photographs of the parts A–C with the compressing directions (yellow arrows) and parts D–G. c,d) Photographs of the sponge devices (c) and colorimetrically estimated *P* (d) with the compression contacting on the parts A–C (sample size: *n* = 3). e,f) Photographs of the sponge devices (e) and colorimetrically estimated *P* (f) with the different contacting parts D–G and compressing directions K–M. Each compression experiment was performed using three different devices (sample size: *n* = 3).

Unknown weak compression stresses in local area were visualized and measured using the PDA/DL sponge device (**Figure** **4**). Unknown compression stresses were applied to the device (15 × 10 × 5 mm) using an ornament of giant panda (Figure 4a). The local compression stress and its distribution applied by irregular‐shaped objects are not easily estimated because the contact area is not exactly measured. Our sponge device directly visualizes and measures the local compression stresses. The sponge devices were compressed by the panda with contacting the different parts A–C in the directions indicated by the yellow arrows in Figure 4b until the balance under the device displayed 1550 g. The compression via each part was performed using three different devices to ensure the reproducibility (Figure 4c,d; Figure S10 and Table S7, Supporting Information). The applied compression stresses were estimated from ∆*x* on the cross‐section of the device using the standard curve in Figure 3e. The measured *P* was 37.26 ± 3.16 kPa for the part A (ear), 15.63 ± 0.37 kPa for the part B (nose), and 16.79 ± 10.31 kPa for the part C (hip and tail) (Figure 4d; Figure S13 and Table S7, Supporting Information). The standard deviation of the part C was larger than that of the other parts because the same contacting state was not achieved by the shape including rounded hip and sharpened tail. Figure 4d indicates that the larger *P* was applied by the more sharpened parts.

The sponge devices were set under the four foots of the panda (the parts D–G in Figure 4a,b). As this object has the different contact states for each foot (Figure 4b), the applied compression stresses are different. The compression stresses were applied in the directions K–M with contacting on the parts D–G using a tester until the force displayed 45 N (Figure 4e). The compression experiment was performed three times using the different devices. The colorimetrically estimated *P* was different for the different contacting states D–G and compressing directions K–M (Figure 4e,f; Figure S13 and Table S7, Supporting Information). When the front part was compressed in the direction M, the larger *P* was observed on the devices under the parts F and G compared with the parts D and E (purple in Figure 4f). On the other hand, when the compression was applied in the direction K, the compression stresses were detected on the devices under the parts D and E (green in Figure 4f). In this manner, the different local compression stresses were visualized and measured by the sponge device.

### 2.5 Imaging of Compression–Stress Distribution

**Figure 5:**
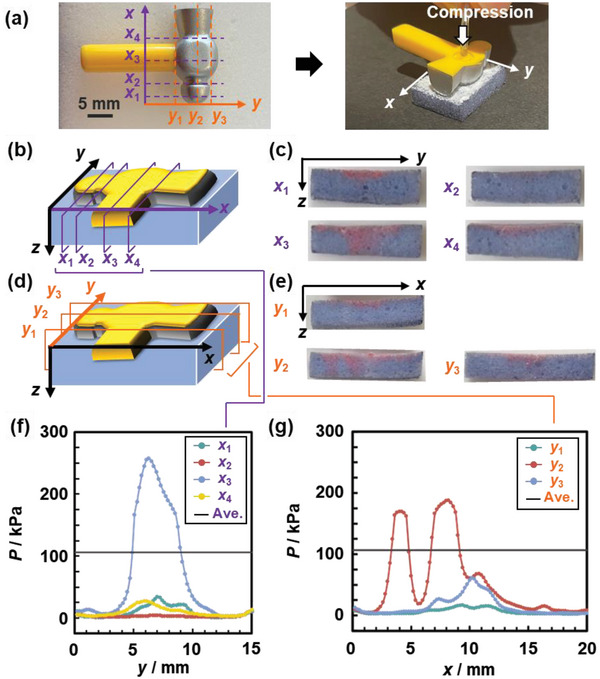
Imaging the compression–stress distribution. a) Photographs of the compressing hammer‐shaped object (left) and positional relation with the PDA/DL sponge device (right). b,c) Schematic illustration (b) and photographs (c) for the analysis of the compression–stress distribution on *y–z* plane at the cross section *x* = *x*_1_, *x*_2_, *x*_3_, and *x*_4_. d,e) Schematic illustration (d) and photographs (e) for the analysis of compression–stress distribution on the *x*–*z* plane at the cross section *y* = *y*_1_, *y*_2_, and *y*_3_. f) Compression–stress distribution on the *y*–*z* plane at *x* = *x*_1_, *x*_2_, *x*_3_, and *x*_4_. g) Compression–stress distribution on the *x–z* plane at *y* = *y*_1_, *y*_2_, and *y*_3_. The average value in the panels (f,g) (black horizontal line) was measured using a scale (Note: running average of the five neighboring plots in the *y* and *x* axes).

The sponge device afforded imaging of compression–stress distribution (**Figure** **5**). The irreversible color change of PDA fixed the applied stresses on each position. The PDA/DL sponge device was compressed for 10 s using a plastic toy of hummer ≈1.1 g in weight and 20 mm in size (Figure 5a). The cross‐sectional images were obtained at the different positions by cutting the device (Figure 5b–e). Here the *y*–*z* (*x* = *x*_1_, *x*_2_, *x*_3_, *x*_4_) and *x*–*z* (*y* = *y*_1_, *y*_2_, *y*_3_) planes were extracted by cutting the device (Figure 5b,d). The ∆*x* values were converted to *P* using the standard curve in Figure 3e. *P* was colorimetrically estimated from the divided areas 0.2 × 5 mm in the *y*–*z* and *x*–*z* planes and the running average of the five neighboring data was plotted in the profiles (Figure 5c,e–g). The compression–stress distribution was visualized by the relationship between the position (*y* or *x*) and *P* (Figure 5f,g). The average compression stress was calculated to be 106.5 kPa from the weight and approximated contact area (horizontal line in Figure 5f,g). For example, the large stress was applied by the convex part in the range of *y* = 5–10 mm at the cross‐section *x* = *x*_3_ in *y*–*z* plane (Figure 5a,b,f), whereas the concave part applied no stresses at the cross‐section *x* = *x*_2_ in *y*–*z* plane. The profiles visualized the stress distribution depending on the shape of the compressing object (Figure 5f,g). In this manner, imaging the compression–stress distribution was achieved by the PDA/DL sponge device.

### 2.6 Softness and Stress‐Distribution Imaging of Simulated Intestinal Tracts Toward Safe Anastomosis

**Figure 6:**
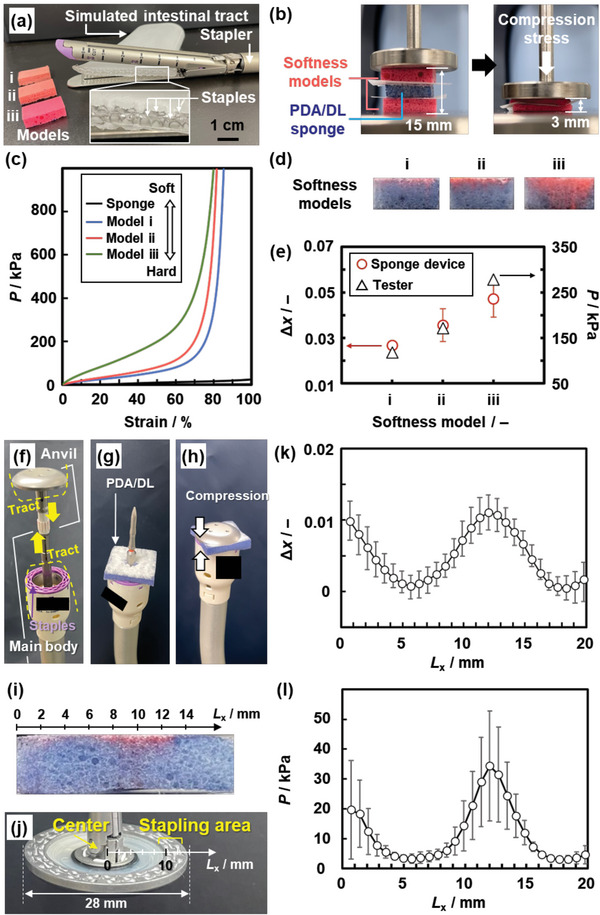
Softness and stress‐distribution imaging for a clinical application. a) Photographs of a simulated intestinal tract with stapling using a linear stapler (right) and softness models i–iii (left). b) Photographs before (left) and after (right) the compression of the PDA/DL sponge device (blue) between the two softness models (red). c) Stress–strain curve of the sponge device (black), softness models i (blue, soft), ii (red, medium), and iii (green, hard). d) Photographs of the sponge device after the compression with the softness models i–iii under the same strain. e) Relationship between the model number and ∆*x* for detection of the softness (sample size: *n* = 3). f–h) Photographs of the automated circular stapling device consisting of anvil and main body (f), setup of the sponge device on the main body (g), and compressing treatment of the sponge between the anvil and main body (h). i) Cross‐sectional photograph of the color‐changed PDA/DL sponge device after the compression. j) Photograph of the backside of the anvil. k) Relationship between *L_x_* and Δ*x* (sample size: *n* = 3). l) Relationship between *L_x_* and *P* with conversion of the Δ*x* value using the standard curve in Figure 3e (sample size: *n* = 3).

The PDA/DL sponge device was applied to study a couple of clinical issues (**Figure** **6**). The number of intestinal cancer patients has increased in recent years. When cancerous segment of intestine is resected, the anastomosis of the remaining tracts is carried out with stapling. For example, the terminal of the cut intestinal tract is compressed and closed with stapling using a linear stapler device (Figure 6a). As the softness and thickness of the tract vary with individuals, the surgeon needs to select an appropriate staple and device. Insufficient stapling may cause anastomotic leakage, leak of the contents of the tracts, leading to serious complications. However, the properties of individual intestinal tract, such as softness and thickness, are not easily estimated from the visual information. In addition, the compression stresses applied by the automated stapling device have impacts on the stapling states. The hard simulated tract actually causes differences in the stapled states (Figure S14, Supporting Information). Our stress imaging sponge device can be applied to study the softness of intestinal tracts and impact of compression stresses caused by the automated surgical devices.

The first target is sensing the softness of the simulated intestinal tracts using the PDA/DL sponge (Figure 6a–e). Softness of the tracts is simulated by the specifically designed three molded rubbers with the different softness (the left part in Figure 6a): models i (soft), ii (medium), and iii (hard). The differences in the softness are characterized by the stress–strain curves to compression stresses (Figure 6c; Figure S15 and Table S8, Supporting Information). The PDA/DL sponge device (15 × 10 × 5 mm) is set between the two models with the same softness (18 × 10 × 5 mm) (left panel in Figure 6b). When the surgeon uses the automated stapling devices, the tract is compressed to the specific thickness and then stapled. Simulating the process, the compression stress is applied using a tester until the thickness of the device and models reach 3 mm (right panel in Figure 6b). The ∆*x* values of the cross sections are measured for each model (Figure 6d). The harder softness models show the larger ∆*x* values, indicating the lager *P* was applied to compress the model to the same thickness (red circles in Figure 6e; Figure S15 and Table S8, Supporting Information). According to the stress–strain curves by the tester, the required *P* is estimated to be 84.3, 128, and 233 kPa for the models i, ii, and iii, respectively (black triangles in Figure 6e). The tester and our device show the same trend (Figure 6e). The results indicate that the sponge device differentiates the softness by the ∆*x* values.

Another target is imaging the stress distribution caused by an automated circular stapling device (Figure 6f–l). During the operation, two tracts guided by the anvil and main body are approached and compressed using the device (Figure 6f). Then, the compressed tracts are connected with the circularly arranged staples and simultaneously punched out with the circular blade. The connection states are influenced by the compression stresses. Here, the stress distribution on the compressed state is visualized and measured using the PDA/DL device. The sponge device is set between the anvil and main body and then compressed using the anastomosis device (Figure 6g,h). The compression stress is released before the stapling. The sponge device is collected and cut to analyze the stress distribution (Figure 6i). The relationship between the position (*L_x_* / mm) and ∆*x* is analyzed on the cross‐section from the center to circumferential direction of the anvil (Figure 6i,j; Figure S16, Supporting Information). The stapling area (8 < *L_x_* < 12) shows the distinct color change to red (Figure 6i,j,k). The ∆*x* is converted to *P* using the standard curve. The applied compression stress on the stapling area is ≈40 kPa (Figure 6l). Our PDA/DL device visualizes and measured the stress distribution. The stress‐distribution imaging can be used in different setting states of the simulated intestinal tracts and types of the stapler devices to study the relationship between the stress distribution and stapling states toward safe anastomosis.

## 3 Conclusion

Two stimuli‐responsive materials of PDA and DL were combined in a commercial melamine sponge. Layered PDA was coated on the fibers in the sponge. DL was set on the surface of the sponge. The color of the sponge device was changed to red with the application of the compression stresses. The compression stress induces disruption of DLs, and then, outflow of the interior liquid. As PEI is diffused into the sponge and intercalated in the interlayer space of PDA, the chemical stress enables the irreversible color change. Although PDA itself shows no color changes with the application of compression stresses, the cascading responses in 3D sponge afford the color changes depending on the strength of weak compression stresses in the range of 3.5–200 kPa. Such weak stresses were not visualized and measured by conventional sensing materials in previous works. The relationship between the applied compression stress and red‐color intensity was used for the colorimetric estimation of unknown compression stresses. The irreversibly color‐changing sponge device visualized compression–stress distribution. Moreover, the sponge device was applied to study a couple of clinical issues, such as the softness of simulated intestinal tracts and the compression–stress distribution applied by an automated anastomosis device. The use of a sponge enables the sensitivity tuning and distribution mapping. The design combining cascading responses and 3D sponge can be applied to fabricate the similar imaging and measuring devices of compression stresses in a variety of strength ranges and length scales.

## Conflict of Interest

The authors declare no conflict of interest.

## Supporting information

Supporting information

## Data Availability

The data that support the findings of this study are available from the corresponding author upon reasonable request.

## References

[1] D. T. Butcher , T. Alliston , V. M. Weaver , Nat. Rev. Cancer 2009, 9, 108. 19165226 10.1038/nrc2544 PMC2649117

[2] M. Prabhune , F. Rehfeldt , C. F. Schmidt , J. Phys. D: Appl. Phys. 2017, 50, 233001.

[3] Y. Huang , X. Fan , S. C. Chen , N. Zhao , Adv. Funct. Mater. 2019, 29, 1808509.

[4] M. Amit , L. Chukoskie , A. J. Skalsky , H. Garudadri , T. N. Ng , Adv. Funct. Mater. 2020, 30, 1905241.

[5] J. Gan , J. Zhang , M. F. Ge , X. Tu , IEEE Sens. J. 2022, 22, 8282.

[6] A. A. Nazari , F. Janabi‐Sharifi , K. Zareinia , IEEE Sens. J. 2021, 21, 8805.

[7] K. Meng , X. Xiao , W. Wei , G. Chen , A. Nashalian , S. Shen , X. Xiao , J. Chen , Adv. Mater. 2022, 34, 2109157. 10.1002/adma.202109357 35044014

[8] S. Pyo , J. Lee , K. Bae , S. Sim , J. Kim , Adv. Mater. 2021, 33, 2005902. 10.1002/adma.202005902 33887803

[9] M. M. Caruso , D. A. Davis , Q. Shen , S. A. Odom , N. R. Sottos , S. R. White , J. S. Moore , Chem. Rev. 2009, 109, 5755. 19827748 10.1021/cr9001353

[10] Y. Sagara , T. Kato , Nat. Chem. 2009, 1, 605. 21378953 10.1038/nchem.411

[11] P. Xue , J. Ding , P. Wang , R. Lu , J. Mater. Chem. C 2016, 4, 6688.

[12] R. Zhang , Q. Wang , X. Zheng , J. Mater. Chem. C 2018, 6, 3182.

[13] Y. Zhuang , R. J. Xie , Adv. Mater. 2021, 33, 2005925. 10.1002/adma.202005925 33786872

[14] Y. Chen , G. Mellot , D. van Luijk , C. Creton , R. P. Sijbesma , Chem. Soc. Rev. 2021, 50, 4100. 33543174 10.1039/d0cs00940g

[15] J. M. Clough , C. Weder , S. Schrettl , Macromol. Rapid Commun. 2021, 42, 2000528. 10.1002/marc.202000528 33210385

[16] W. Qui , J. M. P. Scofield , P. A. Gurr , C. G. Qiao , Macromol. Rapid Commun. 2022, 43, 2100866. 10.1002/marc.202100866 35338794

[17] K. Ariga , Small Methods 2022, 6, 2101577. 10.1002/smtd.202101577 35352500

[18] X. Li , F. Yang , Y. Li , C. Liu , P. Zhao , Y. Cao , H. Xu , Y. Chen , CCS Chem. 2022, 10.31635/ccschem.022.202201874.

[19] V. Kachwal , I. R. Laskar , Top. Curr. Chem. 2021, 379, 28. 10.1007/s41061-021-00341-x 34105028

[20] J. Zhang , B. He , Y. Hu , P. Alam , H. Zhang , J. W. Y. Lam , B. Z. Tang , Adv. Mater. 2021, 33, 2008071. 10.1002/adma.202008071 34137087

[21] Y. Dong , B. Xu , J. Zhang , X. Tan , L. Wang , J. Chan , H. Lv , S. Wen , B. Li , L. Ye , B. Zou , W. Tian , Angew. Chem., Int. Ed. 2012, 51, 10782. 10.1002/anie.201204660 23023926

[22] K. Nagura , S. Saito , H. Yusa , H. Yamawaki , H. Fujihisa , H. Sato , Y. Shimoikeda , S. Yamaguchi , J. Am. Chem. Soc. 2013, 135, 10322. 23815169 10.1021/ja4055228

[23] H. Yang , Z. Sun , C. Lv , M. Qile , K. Wang , H. Cao , B. Zou , Q. Song , Y. Zhang , ChemPlusChem 2018, 83, 132. 31957339 10.1002/cplu.201800080

[24] Y. Liu , Q. Zeng , B. Zou , Y. Liu , B. Xu , W. Tian , Angew. Chem., Int. Ed. 2018, 57, 15670. 10.1002/anie.201810149 30246319

[25] Q. Luo , C. Lv , H. Sheng , F. Cao , J. Sun , C. Zhang , M. Ouyang , B. Zou , Y. Zhang , Adv. Opt. Mater. 2020, 8, 1901836.

[26] H. Yuan , K. Wang , K. Yang , B. Liu , B. Zou , J. Phys. Chem. Lett. 2014, 5, 2968. 26278244 10.1021/jz501371k

[27] Z. Gao , K. Wang , F. Liu , C. Feng , X. He , J. Li , B. Yang , B. Zou , P. Lu , Chem. ‐ Eur. J. 2017, 23, 773. 27796058 10.1002/chem.201604923

[28] D. A. Davis , A. Hamilton , J. Yang , L. D. Cremar , D. V. Gough , S. L. Potisek , M. T. Ong , P. V. Braun , T. J. Martinez , S. R. White , J. S. Moore , N. R. Sottos , Nature 2009, 459, 68. 19424152 10.1038/nature07970

[29] C. E. Diesendruck , B. D. Steinberg , N. Sugai , M. N. Silberstein , N. R. Sottos , S. R. White , P. V. Braun , J. S. Moore , J. Am. Chem. Soc. 2012, 154, 12446. 10.1021/ja305645x 22775564

[30] R. Toivola , P. Lai , J. Yang , S. Jang , A. K. Y. Jen , B. D. Flinn , Compos. Sci. Technol. 2017, 139, 74.

[31] Z. Wang , Z. Ma , Y. Wang , Z. Xu , Y. Luo , Y. Wie , X. Jia , Adv. Mater. 2015, 27, 6469. 26402516 10.1002/adma.201503424

[32] S. Lu , G. Xiao , L. Sui , T. Feng , X. Yong , S. Zhu , B. Li , Z. Liu , B. Zou , M. Jin , J. S. Tse , H. Yan , B. Yang , Angew. Chem., Int. Ed. 2017, 56, 6187. 10.1002/anie.201700757 28378520

[33] M. Andrzejewski , N. Casati , A. Katrusiak , Dalton Trans. 2017, 46, 14795. 29048089 10.1039/c7dt02511d

[34] C. Wu , C. Tu , J. Aimi , J. Zhang , T. Chen , C. Wang , C. Huang , Polym. Chem. 2020, 11, 6423.

[35] M. Liu , Z. Fu , R. Sun , J. Yuan , C. Liu , B. Zou , B. Wang , H. Kou , ACS Appl. Electron. Mater. 2021, 3, 1368.

[36] X. Wang , C. Qi , Z. Fu , H. Zhang , J. Wang , H. Feng , K. Wang , B. Zou , J. w. Y. Lam , B. Z. Tang , Mater. Horiz. 2021, 8, 630. 34821280 10.1039/d0mh01251c

[37] Z. Ding , T. Lu , C. Bi , B. Li , S. T. Zhang , W. Xu , S. Jiang , Mater. Chem. Front. 2021, 6, 86.

[38] W. Yin , Z. Yang , S. Zhang , Y. Yang , L. Zhao , Z. Li , B. Zhang , S. Zhang , B. Han , H. Ma , J. Mater. Chem. 2021, 5, 2849.

[39] B. Poggi , L. Bodelot , M. Louis , R. Metivier , C. Allain , J. Mater. Chem. 2021, 9, 12111.

[40] M. Nakamitsu , K. Oyama , H. Imai , S. Fujii , Y. Oaki , Adv. Mater. 2021, 33, 2008755. 10.1002/adma.202008755 33615567

[41] T. Yamakado , S. Saito , J. Am. Chem. Soc. 2022, 144, 2804. 35108003 10.1021/jacs.1c12955

[42] M. A. Reppy , B. Piindzola , Chem. Commun. 2007, 4317. 10.1039/b703691d 17957278

[43] B. Yoon , S. Lee , J. M. Kim , Chem. Soc. Rev. 2009, 38, 1958. 19551176 10.1039/b819539k

[44] X. Sun , T. Chen , S. Huang , L. Li , H. Peng , Chem. Soc. Rev. 2010, 39, 4244. 20877863 10.1039/c001151g

[45] R. Jelinek , M. Ritenberg , RSC Adv. 2013, 3, 21192.

[46] X. Qian , B. Städler , Chem. Mater. 2019, 31, 1196.

[47] M. Weston , A. D. Tjandra , R. Chandrawati , Polym. Chem. 2020, 11, 166.

[48] F. Fang , F. Meng , L. Luo , Mater. Chem. Front. 2020, 4, 1089.

[49] Y. Oaki , Chem. Commun. 2020, 56, 13069. 10.1039/d0cc05931e 33021619

[50] B. Das , S. Jo , J. Zheng , J. Chen , K. Sugihara , Nanoscale 2022, 14, 1670. 35043814 10.1039/d1nr07129g

[51] S. Lee , J. M. Kim , Macromolecules 2007, 40, 9201.

[52] S. Dei , M. Matsumoto , A. Matsumoto , Macromolecules 2008, 41, 2467.

[53] S. Ampornpun , S. Montha , G. Tumcharern , V. Vchirawongkwin , M. Sukwattanasinitt , S. Wacharasindhu , Macromolecules 2012, 45, 9038.

[54] S. Dolai , S. K. Bhunia , S. S. Beglaryan , S. Kolusheva , L. Zein , R. Jelinek , ACS Appl. Mater. Interfaces 2017, 9, 2891. 28029773 10.1021/acsami.6b14469

[55] Y. Ishijima , H. Imai , Y. Oaki , Chem 2017, 3, 509. 10.1039/c6sc03350d PMC5297333 28451212

[56] G. Shin , M. I. Khazi , U. Kundapur , B. Kim , Y. Kim , C. W. Lee , J. M. Kim , ACS Macro Lett. 2019, 8, 610. 35619370 10.1021/acsmacrolett.9b00169

[57] J. Seo , C. Kantha , J. F. Joung , S. Park , R. Jelinek , J. M. Kim , Small 2019, 15, 1901342. 10.1002/smll.201901342 30968560

[58] N. Phonchai , C. Khanantong , F. Kielar , R. Traiphol , N. Traiphol , ACS Appl. Nano Mater. 2019, 2, 4489.

[59] Y. Mochizuki , H. Imai , Y. Oaki , ChemPlusChem 2021, 86, 1563. 34432949 10.1002/cplu.202100300

[60] N. Shioda , J. M. Heo , B. Kim , H. Imai , J. M. Kim , Y. Oaki , Sens. Diagn. 2022, 1, 160.

[61] B. Tieke , G. Lieser , G. Wegner , J. Polym. Sci., Polym. Chem. Ed. 1979, 17, 1631.

[62] R. A. Nallieheri , M. F. Rubner , Macromolecules 1991, 24, 517.

[63] R. W. Carpick , D. Y. Sasaki , A. R. Burns , Langmuir 2000, 16, 1270.

[64] S. Chae , J. P. Lee , J. M. Kim , Adv. Funct. Mater. 2016, 26, 1769.

[65] V. K. Rao , N. Shauloff , X. M. Sui , H. D. Wagner , R. Jelinek , J. Mater. Chem. C 2020, 8, 6034.

[66] Q. Li , Y. X. Wang , Y. Chen , ACS Macro Lett. 2022, 11, 103. 35574789 10.1021/acsmacrolett.1c00715

[67] L. Juhasz , R. D. Ortuso , K. Sugihara , Nano Lett. 2021, 21, 543. 33284635 10.1021/acs.nanolett.0c04027

[68] K. Kido , T. Sumoto , Y. Yasui , Y. Nakamura , S. Fujii , Adv. Powder Technol. 2017, 28, 1977.

