# Supplementary material for: Stimuli‐Responsive Sponge for Imaging and Measuring Weak Compression Stresses
Source: Adv Sci (Weinh). 2022 Dec 11;10(3):2206097. doi: 10.1002/advs.202206097 (PMC9875629; doi:10.1002/advs.202206097)
Supplement: Supplementary file 1 — Supporting information [file ADVS-10-2206097-s001.pdf]

Supporting Information

Stimuli-Responsive Sponge for Imaging and Measuring Weak Compression Stresses

Nahoko Ono,[a] Ryo Seishima,[b] Koji Okabayashi,[b] Hiroaki Imai,[a] Syuji Fujii,*[,c] Yuya Oaki*[,a]

[a] Department of Applied Chemistry, Faculty of Science and Technology, Keio University, 3-14-1 Hiyoshi, Kohoku-ku, Yokohama 223-8522, Japan.

[b] Department of Surgery, School of Medicine, Keio University, 35 Shinanomachi, Shinjuku-ku, Tokyo 160-8582

[c] Department of Applied Chemistry, Faculty of Engineering, Osaka Institute of Technology, 5-16-1 Omiya, Asahi-ku, Osaka 535-8585, Japan

*E-mail: syuji.fujii@oit.ac.jp, oakiyuya@applc.keio.ac.jp

Contents

Experimental methods (Scheme 1)	P. S2
Previous works about detection of compression stresses (Figure S1)	P. S5
Structural data of melamine sponge and PDA coating (Figure S2 and Table S1)	P. S6
Structural data of DL (Figure S3 and Table S2)	P. S7
Structure characterization of the color-changed device (Figure S4)	P. S9
Influence of the processes with the compression (Figure S5)	P. S11
Photographs of the compressed sponge devices (Figure S6 and Table S3)	P. S12
Sensitivity tuning by changes of the melamine sponges (Figure S7 and Table S4)	P. S13
Sensitivity tuning by changes in the loaded amount of DL (Figure S8 and Table S5)	P. S14
Response to applied duration of the compression stresses (Figure S9)	P. S16
Response of DLs introduced in the inside of the sponge (Figure S10)	P. S17
Distribution of the fragmented SiO_2 particles in the sponge (Figure S11)	P. S18
Reference experiments using the colored DL (Figure S12 and Table S6)	P. S19
Application of unknown *P* (Figure S13 and Table S7)	P. S21
Stapling states of the softness models I–III (Figure S14)	P. S23
Softness sensing of the models I–III (Figure S15 and Table S8)	P. S24
Stress-distribution imaging using a circular stapler (Figure S16)	P. S25

Experimental methods

Preparation of the PDA/DL sponge device. Two types of commercially available melamine sponges (20 × 15 × 5 mm) were dipped in 4 cm^3 of the precursor acetone solution containing 10 mg cm^{-3} 10,12-pentacosadiynoic acid (PCDA, TCI, 97.0%) for 10 min. The PCDA-coated sponge was dried at room temperature. UV light (6 W, As-one Handy UV lamp SLUV-6, 254 nm) was irradiated from both the top and bottom sides for each 30 sec and four lateral sides for each 15 sec to obtain the PDA-coated sponge. DL was prepared according to our previous reports.[40,68] Aqueous solution containing 20 wt% polyethyleneimine (PEI, Nippon Shokubai, branched chain with $\overline{M}_n = 300$) was prepared with purified water. The PEI solution (25 cm^3) and silica particles (2.78 g) (PDMS-SiO_2, Aerosil, RY-300, 21–27 nm in diameter) were mixed at 20000 rpm for 5 s using a mixer (Lab Mill, Osaka Chemical Company). DL in the range of 105 and 250 μm was collected using stainless sieves with the pore sizes in 105 and 250 μm. DL was stored in a sealed polypropylene bottle until the use. The resultant DL, ca. 10 mg, was homogeneously dispersed on the top surface of the PDA-coated sponge. The colored DL as a reference sample was prepared with solution containing 10^{-3} mol dm^{-3} rhodamine B (Kanto).

Structural characterization. Macroscopic and microscopic structures of DLs and the sponge devices were observed by optical microscopy (OM, Keyence VHX-1000) and scanning electron microscopy (SEM, JEOL JSM-7100) operated at 5.0 V. The disruption behavior of DLs in the device was analyzed by X-ray computer tomography (XCT, Shimadzu, XDimensions 300) and SEM (Carl-Zeiss Merlin VP compact) with EDX equipment. The polymerization behavior was analyzed by Raman spectroscopy (Renishaw, In-via Raman) with the excitation light 785 nm. The intercalation state of PEI in the layered PDA was studied by Fourier-transform infrared spectroscopy (FT-IR, Jasco, FT/IR-4200) and X-ray diffraction (XRD, Bruker, D8 Advance).

Imaging and measuring the compression stresses. The overall procedure was shown in Scheme S1. The sponge device was used in 10 s after the dispersion of DLs. Compression stresses were applied on the PDA/DL sponge device, typically 15 × 10 × 5 mm, using a table-top tensile tester (Shimadzu EZ-LX). The force in the range of 0.05–45 N was applied to the sponge device via a metal probe (contact area, 150 mm^2). The compression stresses correspond to 0.35–675 kPa. The device size was 15 × 10 × 5 mm for the range of 0–321 kPa and 10 × 7.5

× 5 mm for 541 and 675 kPa. The contact area was determined by the size of the device. After the compression stresses were applied for 10 s, the remaining DL was immediately removed with air blow. The sponge device was cut by tweezers to prepare the cross section. The photograph was taken using a smart phone (iPhone 13) at 100 s after releasing the compression stress. The red-color intensity (x) values were calculated from the RGB values of the cross sections (10 × 5 mm) using a software for image analysis (Image J) according to Eqs, S1 and S2.[S1] The successive processes were completed within total 120-150 s.

$$\begin{bmatrix} X \\ Y \\ Z \end{bmatrix} = \begin{bmatrix} 0.4124 & 0.3576 & 0.1805 \\ 0.2126 & 0.7152 & 0.0722 \\ 0.0193 & 0.1192 & 0.9505 \end{bmatrix} \begin{bmatrix} R \\ G \\ B \end{bmatrix} \quad \ldots \text{(Eq. S1)}$$

$$(x, y) = \left(\frac{X}{X+Y+Z}, \frac{Y}{X+Y+Z}\right) \quad \ldots \text{(Eq. S2)}$$

The data including the mean and standard deviation were obtained using three different sponge devices to ensure the reproducibility. In the present work, the thickness of the device was constant at 5 mm. As Δx value is an increment of the ratio of the red-color domain to the original state ($\Delta x = x - x_0$), the relationship between P and Δx in Figure 3e is used regardless of the lateral size in the cross section of the device.

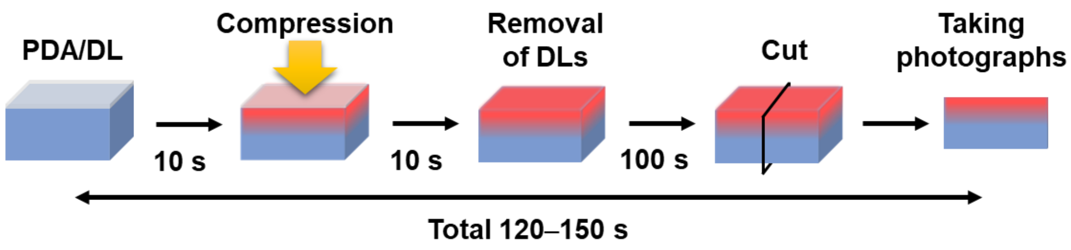

Scheme 1. Schematic illustration of the procedure and time course from the device preparation of PDA/DL, compression, removal of the remaining DLs, cutting of the sponge to prepare the cross section, and taking photographs for the readout Δx.

Statistical analysis. All the colorimetric analyses of the compression stresses in Figures 3, 4, and 6 were performed using three different devices (sample size: n = 3). A set of the representative photographs was displayed in the main text. The other photographs were listed in the Supporting Information. The original numerical data were summarized in the Supporting

Information. The mean and standard deviation were displayed in Figures 3, 4 and 6 and mentioned in the main text.

Additional Reference

[S1] E. Reinhard, W. Heidrich, P. Debevec, S. Pattanaik, G. Ward, K. Myszkowski, High Dynamic Range Imaging: Acquisition, Display, and Image-Based Lighting (2nd edition), Elsevier Science, Saint Louis, **2010**, Chapter 2, P. 35.

Previous works about detection of compression stresses

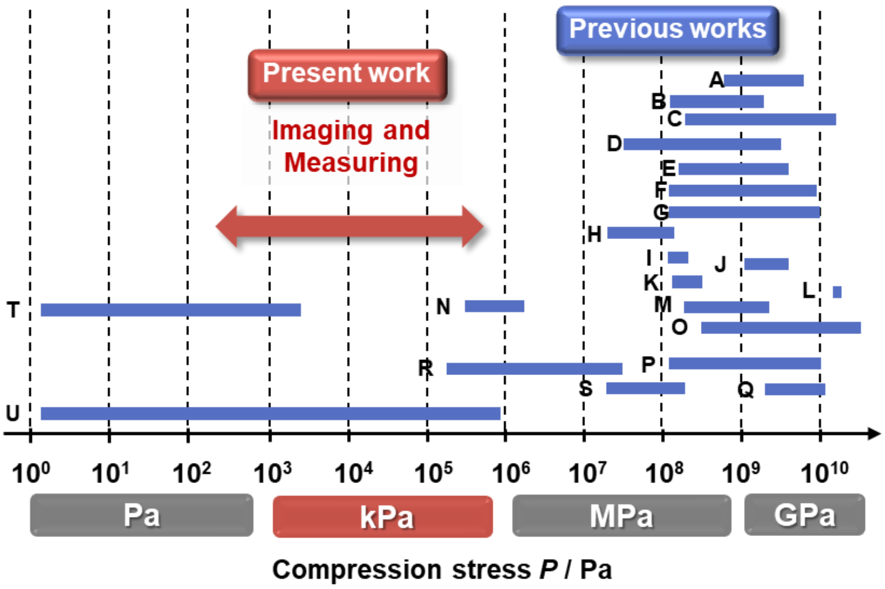

Figure S1. Detection ranges of the compression-stress sensing materials in previous works. The symbols A–U correspond to the references [21–41], respectively.

The compression stresses in the range of 10^3–10^5 Pa order were not detected in previous works about mechano-responsive materials in 2021.[21–40] A recent paper published in 2022 showed the fluorescent color changes in 10^0–10^6 Pa.[41] However, the specific mechanophore needs an exitation light to detect the stress. Our present device directly showed the visible color change in response to the stress in the range of 10^3–10^5 Pa order.

Structural data of melamine sponge and PDA coating

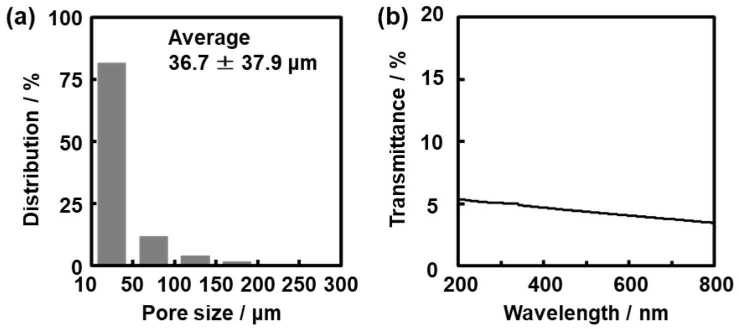

Figure S2. Pore-size distribution (a) and UV-Vis transmittance spectrum (b) of a commercial melamine sponge used in the present work.

Table S1. PDA coating in the melamine sponge.

Entry	Weight / mg		
	Melamine sponge	PDA/ Melamine sponge	PDA
(i)	14.09	16.36	2.27
(ii)	13.85	16.15	2.30
(iii)	13.03	15.43	2.40
Average	13.66	15.98	2.32

The average size and distribution of the pore were estimated from the SEM images. The average width of the fibers in the network was 4.72 ± 1.82 μm. The melamine sponge (20 × 15 × 5 mm) contained average 17 wt% of PDA to its own weight (Table S1). UV light permeated the melamine sponge (20 × 10 × 2.5 mm) ca. 5 % at 254 nm (Figure S2b). The thickness of the sponge was reduced to half. As UV light was irradiated to both the surface and back sides of the sponge, UV transmission to 2.5 mm in depth supports the irradiation to whole of the sponge.

Structural data of DL

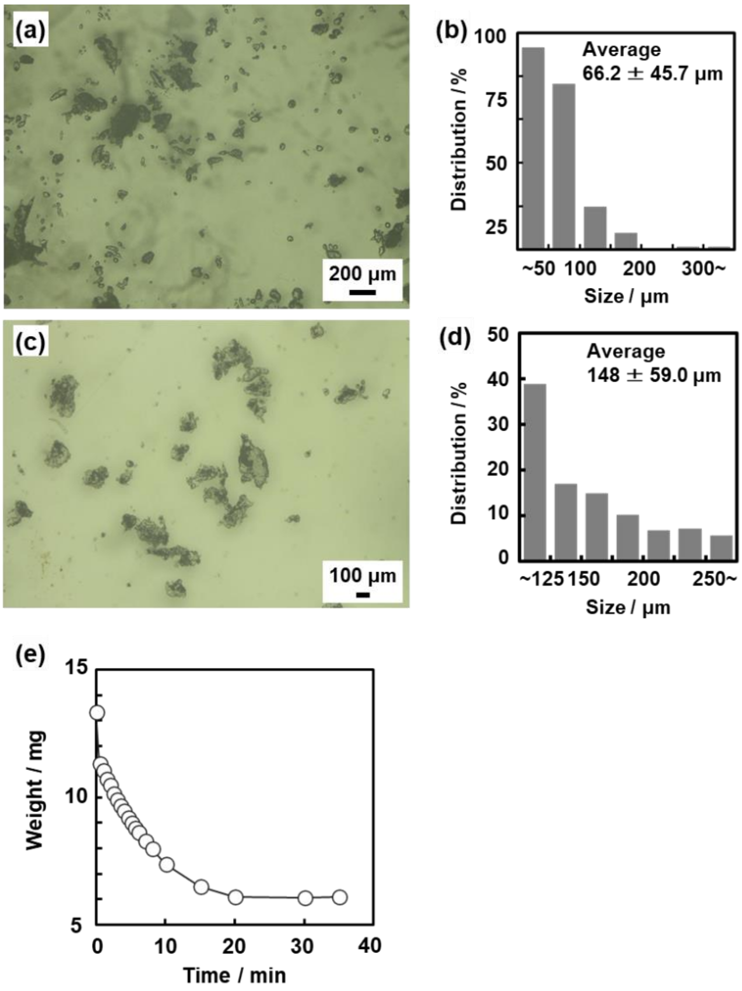

Figure S3. Particle-size distribution (a–d) and time-dependent weight variation (e) of DLs after the preparation (a,b) and subsequent size selection using stainless sieves 125 and 250 μm in the pore diameter (c,d). (a,c) Optical microscopy images. (b,d) Particle-size distributions and their averages based on the optical microscopy images. (e) Relationship between the time and weight of DL maintained on a chartula at room temperature under ambient pressure.

Table S2. Relationship between the particle size of DL and weight content of PEI solution.

DL Size / μm	–53	53–125	125–180	180–212	212–250	250–300	300–
Weight of PEI solution in 1 mg of DL / mg	0.418	0.121	0.0785	0.0632	0.0637	0.0459	0.0606
Weight percentage of PEI solution / %	58.2	87.9	92.1	93.7	93.6	95.4	93.9

In the present work, the size-selected DLs were used for preparation of the devices (Figure S3c,d), even though DLs smaller than 125 μm and lager than 250 μm were included after the sieving. The smaller and larger DLs were removed by the particle-size selection (the colored data in Table S2).

Water as the solvent of the interior liquid in DL was gradually evaporated within ca. 20 min at room temperature under ambient pressure (Figure S3e). On the other hand, the resultant DL was stored in a plastic bottle after the preparation until the use. When the PDA/DL sponge device was prepared, a specific amount of DL was taken from the storage bottle. The disruption of DLs with the evaporation of water was not observed in a plastic bottle. In contrast, DL stored in a glass bottle caused the disruption with evaporation of water. When water evaporated from DL forms the liquid film with wetting on the hydrophilic surface of glass, the interior liquid as the smaller water droplets diminishes by Ostwald ripening. DLs are preserved in a plastic bottle because the hydrophobic wall prevents water from wetting on the wall. In this manner, the stored DL in a plastic bottle can be used anytime.

Structure characterization of the color-changed device

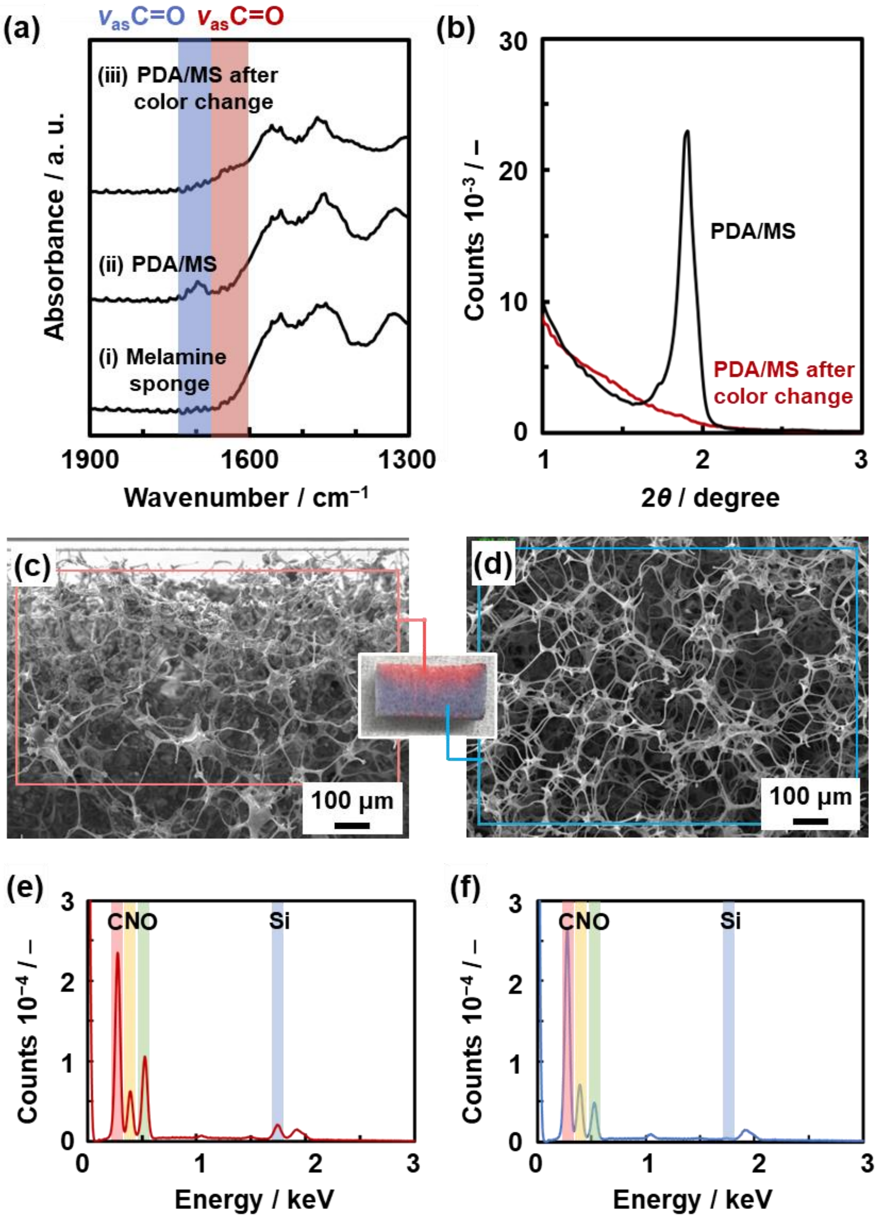

Figure S4. Microscopic and macroscopic structural analyses of the color-changed PDA/DL sponge device. (a,b) FT-IR spectra (a) and XRD patterns (b) of a melamine sponge (MS) (i), PDA-coated MS (ii), and PDA-coated MS after the color changes with the disruption of the loaded DL (iii). (c,d) Cross-sectional SEM images and its EDX spectra of the red (c,e) and remaining blue (d,f) parts in the sponge device (the middle photograph).

Whereas the original PDA on the melamine sponge showed the absorption peak corresponding to dimerized carboxy groups of C=O around 1690 cm^{-1}, the absorption peak corresponding to

carboxylate groups of C=O appeared around 1650 cm^{-1} after the disruption of DL (Figure S4a). The peak characteristic to the layered structure at $2\theta = 1.90$ ° ($d = 4.64$ nm) was weakened and shifted to $2\theta = 1.45$ ° ($d = 6.09$ nm). An increase in the d value is consistent with that of the PEI-intercalated layered PDA in our previous work.[S2]

SEM image and EDX analysis indicate the presence of nitrogen (N) and silicon (Si) originating from the interior PEI and shell SiO_2 particles in the red-colored domain, respectively (Figure S4c,e). In contrast, these elements were not detected in the blue-colored domain (Figure S4d,f). These facts indicate infiltration of the fragmented SiO_2 particles and interior PEI in the sponge.

Additional Reference

[S2] K. Watanabe, H. Imai, Y. Oaki, *J. Mater. Chem. C* **2020**, *8*, 1265.

Influence of the processes with the compression

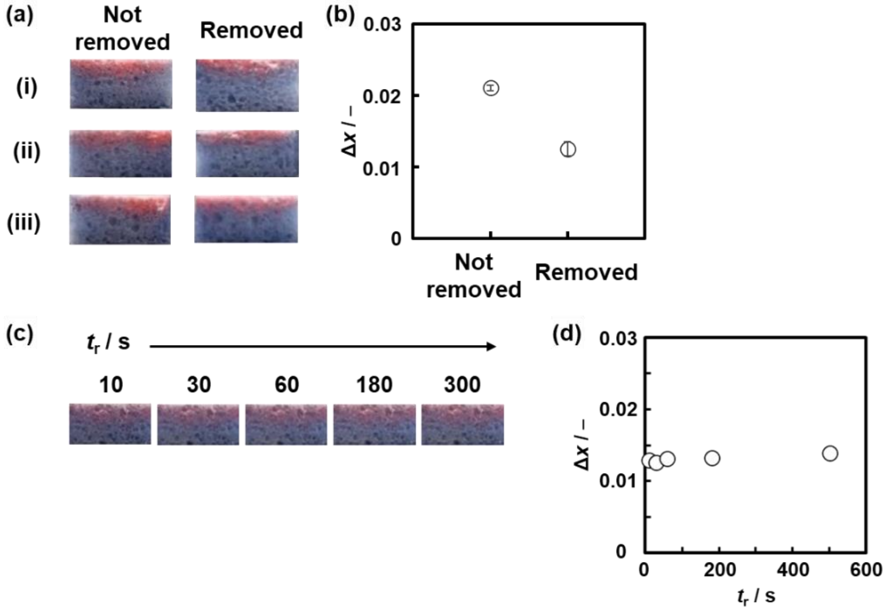

Figure S5. Changes in the duration of the compression (a,b) and time for the readout of Δx (c,d). (a) Photographs of the PDA/DL sponge device with and without removal of the remaining DL before cutting the device to prepare the cross section after the compression (P = 3.3 kPa). (b) Summary of Δx in the panel (a). (c) Photographs of the sponge device after the compression (P =100 kPa) and cutting the device with changing the time for the readout (t_r). (d) Relationship between t_r and Δx.

After applying stress, the remaining DL was removed from the sponge device. The device was cut by scissors to obtain the cross section for taking the photographs. When the DL was not removed, the cutting caused the further disruption leading to the color change (Figure S5). Therefore, the remaining DLs were removed by air blow. A commercial small handy blower for cleaning of camera and lens was used to this removal process. Propellant or high-pressure gas are not required. In addition, t_r has no influence on Δx.

Photographs of the compressed sponge device

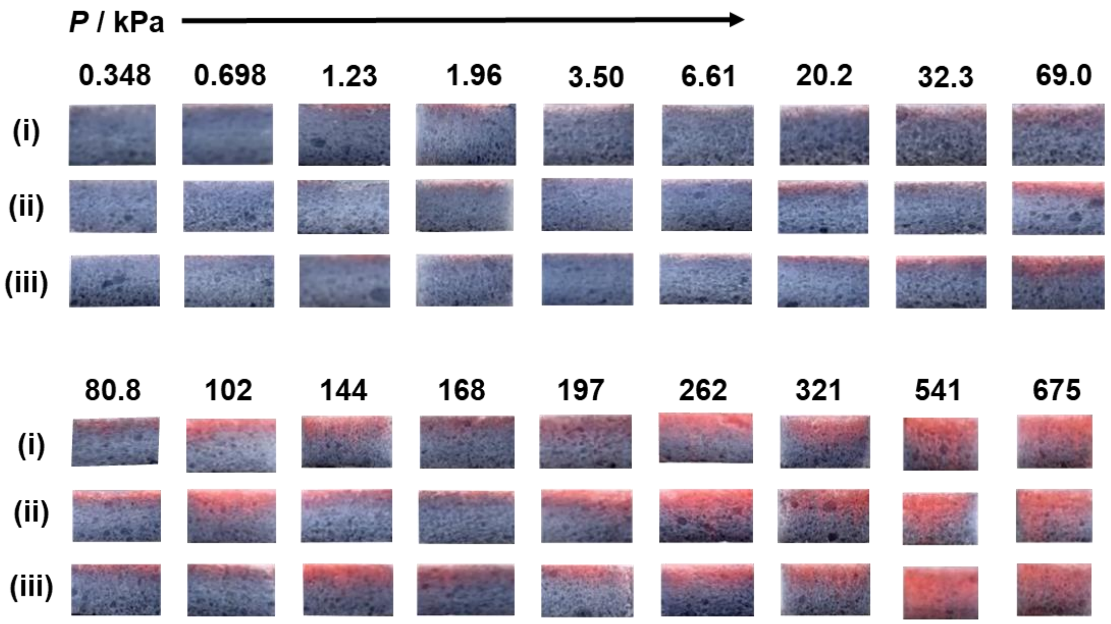

Figure S6. Cross-sectional photographs of the PDA/DL sponge device with the application of the compression stresses on three different devices (i)-(iii) using a tester.

Table S3. Summary of P and Δx. (average: $\overline{\Delta x}$, S. D.: standard deviation)

Ideal P / kPa	0.348	0.698	1.23	1.96	3.50	6.61	20.2	33.4	69.0
(i) Δx / –	0.00168	-0.0012	0.00245	0.00863	0.00477	0.00244	0.00550	0.0163	0.00892
(ii) Δx / –	0.0000475	0.00368	0.00290	0.00590	0.00210	0.00657	0.00867	0.0194	0.0172
(iii) Δx / –	0.00198	0.00455	0.00392	0.00341	-0.00150	0.00616	0.00481	0.00990	0.0110
$\overline{\Delta x}$ / –	0.00121	0.00234	0.00309	0.00598	0.00180	0.00499	0.00633	0.0145	0.0124
S. D.	0.000895	0.00312	0.000751	0.00261	0.00314	0.0239	0.00206	0.00592	0.00428

Continued

Ideal P / kPa	80.8	102	144	168	221	264	321	541	675
(i) Δx / –	0.00921	0.0254	0.0137	0.0131	0.0247	0.0322	0.0308	0.0485	0.0488
(ii) Δx / –	0.0168	0.0204	0.0153	0.0222	0.0289	0.0402	0.0336	0.0546	0.0529
(iii) Δx / –	0.0132	0.0160	0.0195	0.0119	0.0216	0.0285	0.0339	0.0617	0.0491
$\overline{\Delta x}$ / –	0.0131	0.0206	0.0184	0.0157	0.0251	0.0336	0.0327	0.0549	0.0503
S. D.	0.00380	0.00467	0.00514	0.00565	0.00367	0.00594	0.00171	0.00659	0.00228

Sensitivity tuning by changes of the melamine sponges

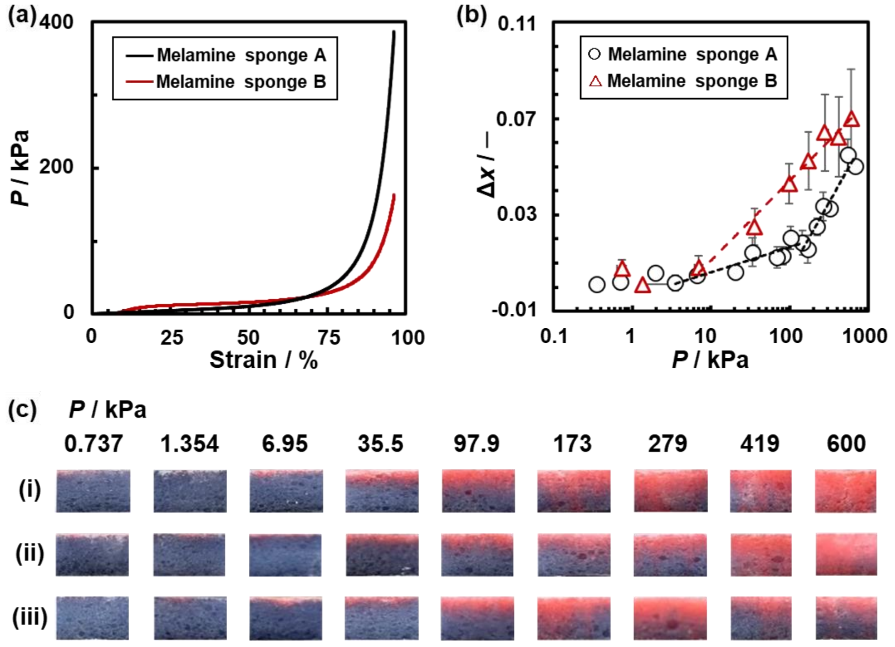

Figure S7. Cross-sectional photographs of the PDA/DL device with changes of the melamine sponge with different softness. (a) Stress-strain curves of the melamine sponges A (used as the standard in the present work) and B (the different softness). (b) Relationship between P and Δx of the devices based on the sponges A and B. (c) Photographs of the three different devices (i)–(iii) based on the sponge B with the application of the compression stresses.

Table S4. Summary of P and Δx.

Ideal P / kPa	0.737	1.354	6.95	35.5	97.9	173	279	419	600
(i) Δx / –	0.0083	0.00091	0.0116	0.0284	0.0523	0.0607	0.0743	0.0657	0.0889
(ii) Δx / –	0.00111	0.00208	0.00263	0.0306	0.0370	0.0386	0.0459	0.0771	0.0731
(iii) Δx / –	0.00406	0.00024	0.0104	0.0164	0.0393	0.0580	0.0722	0.0443	0.0481
$\overline{\Delta x}$ / –	0.00783	0.00108	0.00821	0.00251	0.0429	0.0524	0.0641	0.0624	0.0700
S. D.	0.00355	0.000933	0.00487	0.00762	0.00824	0.00120	0.0158	0.00167	0.00205

The responsivity was improved using the more elastic melamine sponge B compared with the sponge A. As the sponge B is strained by the weaker compression stress, the diffusion distance of PEI into the sponge is shortened.

Sensitivity tuning by changes in the loaded amount of DL

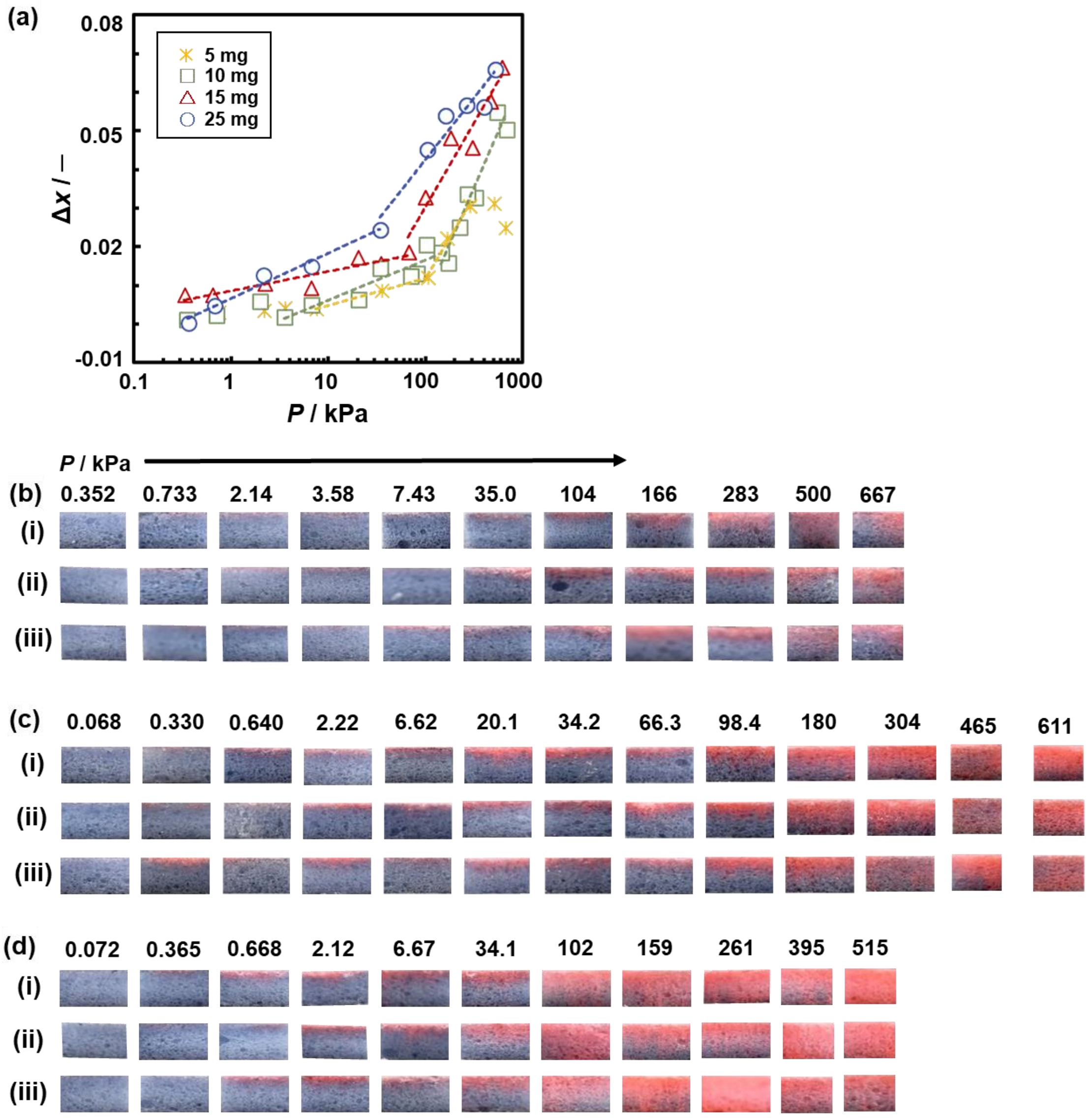

Figure S8. Cross-sectional photographs of the PDA/DL sponge loaded with the different weight of DL. (a) Relationship between *P* and Δ*x* of the devices with the loading of DL 5 (yellow crosses), 10 (green squares, standard in the present work), 15 (red triangles), and 25 (blue circles) mg. (b–d) Photographs of the three different devices with loading of DL 5 (b), 15 (c), and 25 (d) mg with the application of the compression stresses.

Table S5. Summary of P and Δx values.

DL / mg	5										
Ideal P / kPa	0.352	0.733	2.14	3.58	7.43	35.0	104	166	283	500	667
(i) Δx / –	0.000812	0.00284	0.00362	0.00297	0.00237	0.09661	0.0879	0.0214	0.0200	0.0275	0.0289
(ii) Δx / –	0.00157	0.00294	0.000283	0.00491	0.00638	0.0121	0.0108	0.0193	0.0296	0.0273	0.0219
(iii) Δx / –	0.00181	0.00329	0.0104	0.00448	0.00296	0.00721	0.0164	0.0256	0.0416	0.0389	0.0235
$\overline{\Delta x}$ / –	0.00140	0.00302	0.00347	0.00412	0.00391	0.00865	0.0120	0.0221	0.0304	0.0312	0.0248
S. D.	0.000524	0.000239	0.00311	0.00102	0.00217	0.00302	0.0395	0.00319	0.0108	0.00662	0.00368

DL / mg	15										
Ideal P / kPa	0.0675	0.330	0.640	2.22	6.62	20.1	34.2	66.3	98.4	180	304
(i) Δx / –	-0.00210	0.00575	0.00291	0.00898	0.00597	0.0208	0.0122	0.0152	0.0312	0.0525	0.0669
(ii) Δx / –	0.00286	0.0135	0.00949	0.0126	0.0125	0.0136	0.0184	0.0227	0.0298	0.0485	0.0381
(iii) Δx / –	0.00251	0.00304	0.00986	0.00957	0.00949	0.0172	0.0164	0.0177	0.0374	0.0434	0.0580
$\overline{\Delta x}$ / –	0.00109	0.00742	0.00742	0.0104	0.00933	0.0172	0.0157	0.0185	0.0328	0.0481	0.0456
S. D.	0.00276	0.00540	0.00391	0.00196	0.00327	0.00359	0.0319	0.00386	0.00402	0.00452	0.0212

Continued

Ideal P / kPa	465	611
(i) Δx / –	0.0601	0.0734
(ii) Δx / –	0.0634	0.0670
(iii) Δx / –	0.0492	0.0586
$\overline{\Delta x}$ / –	0.0576	0.0663
S. D.	0.00746	0.00742

DL / mg	25										
Ideal P / kPa	0.0717	0.356	0.668	2.12	6.67	34.1	102	159	261	395	515
(i) Δx / –	0.00224	0.0011	0.00569	0.0134	0.0165	0.0246	0.0435	0.0416	0.0630	0.0496	0.0574
(ii) Δx / –	0.00272	0.00051	0.00713	0.0121	0.0146	0.0234	0.0540	0.0564	0.0450	0.0621	0.0759
(iii) Δx / –	0.00119	0.00156	0.00000261	0.0127	0.0140	0.0254	0.0378	0.0636	0.0617	0.0569	0.0654
$\overline{\Delta x}$ / –	0.00205	0.000325	0.00474	0.0128	0.0150	0.0245	0.0451	0.0539	0.0566	0.0562	0.0659
S. D.	0.000781	0.00134	0.00411	0.000634	0.00130	0.00105	0.00822	0.0112	0.0100	0.00630	0.00870

The responsivity was improved with an increase in the amount of the loaded DL. As the number of collapsed DLs and volume of the released PEI increases, the responsivity is improved.

Response to applied duration of the compression stresses

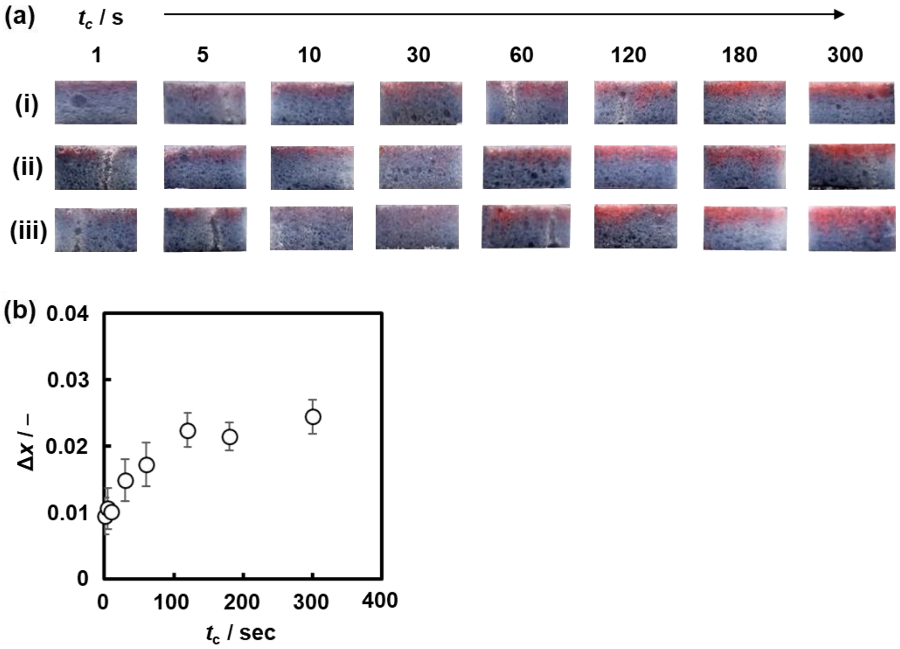

Figure S9. Color-change properties in response to the applied duration (t_c) of the compression stresses. (a) Cross-sectional photographs of the device with the application of the compression stresses with changing t_c of the compression stress 66.7 kPa. (b) Relationship between t_c and Δx.

The PDA/DL device showed the color changes in response to t_c. As the diffusion length of the interior liquid increased with increasing t_c, the larger Δx is observed.

Response of DLs introduced in the inside of the sponge

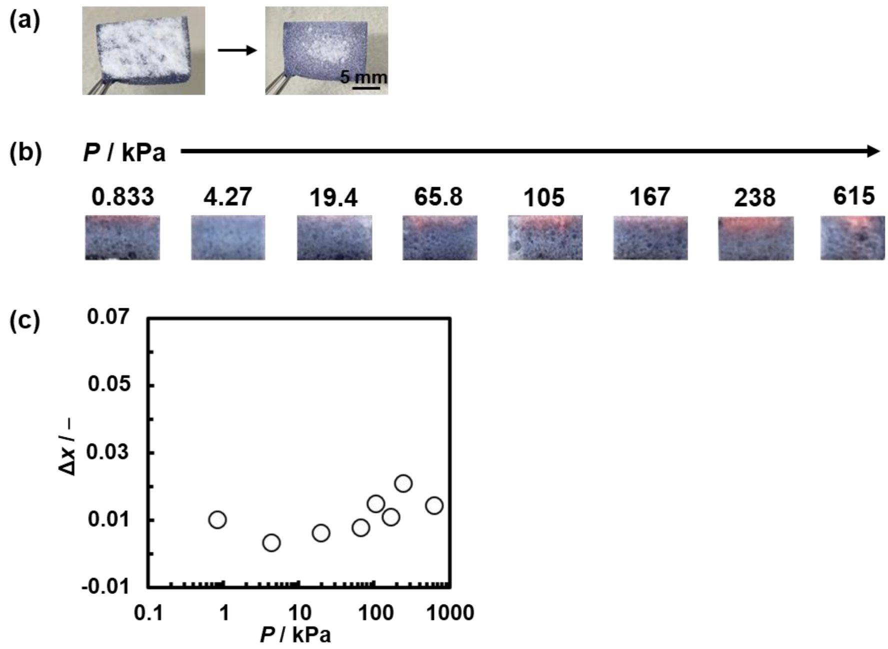

Figure S10. PDA sponge device with DLs introduced in the inside of the sponge. (a) Photographs of the DLs (53–125 μm) dispersed on the surface of the sponge (left) and subsequent introduction into the inside of the sponge with the shaking (right). (b) Cross-sectional photographs of the device with the application of the compression stresses. (c) Relationship between P and Δx.

The DL was introduced in the sponge with shaking (Figure S8a). In this reference experiment, the particle size of DLs was changed from 125–250 μm (normal condition) to 53–125 μm to introduce in the inside of the sponge. When the PDA sponge including the DLs in the inside of the sponge, the colorimetric response was not observed (Figure S9b,c). However, the detailed reasons why DLs in the inside of the sponge device are not disrupted in response to the stress are still unclear. We are now studying the detailed disruption mechanisms of DLs.

Distribution of the fragmented SiO_2 particles in the sponge

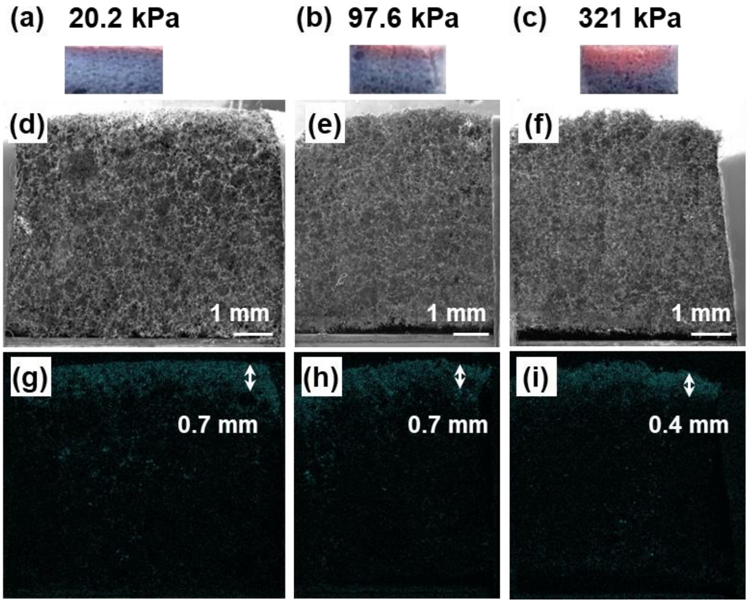

Figure S11. Distribution of the fragmented SiO_2 particles in the sponge with the application of the compression stresses P = 20.2, 97.6 and 321 kPa. (a–c) Cross-sectional photographs. (d–f) SEM images. (g–i) EDX mapping of Si (green) on the SEM images.

Disruption of DLs leads to dispersion of the fragmented SiO_2 particles in the sponge. The dispersion behavior of the fragmented SiO_2 particles was not different by changes in P. The fact indicates that DLs disrupt near the surface of the sponge.

Reference experiments using the colored DL (c-DL)

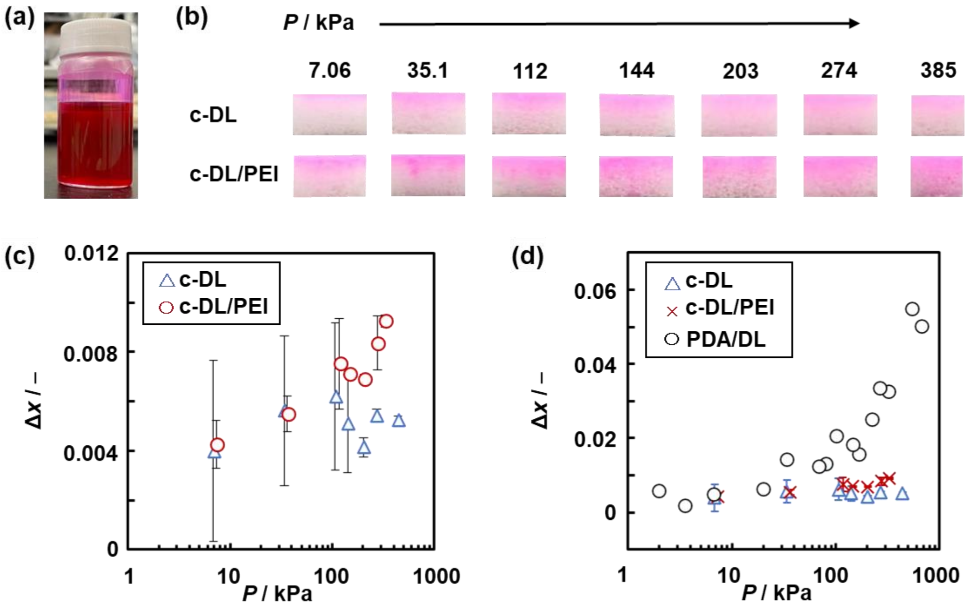

Figure S12. Diffusion depth of PEI in the sponge. (a) Photograph of 10^{-3} mol dm^{-3} rhodamine B solution for preparation of c-DLs. (b) Cross-sectional photographs of the white sponge after disruption of c-DL and c-DL/PEI with the compression. (c) Relationship between P and Δx of the reference sponge devices with loading c-DL and c-DL/PEI. (d) Comparison of the PDA/DL device with the reference devices.

Table S6. Summary of P and L_{red}.

Device	Colored DL without PEI						
Ideal P / kPa	6.93	35.8	105	140	209	267	407
(i) L_{red} / mm	1.077	1.000	1.615	1.308	1.154	1.077	1.000
(ii) L_{red} / mm	0.538	1.000	1.156	1.154	1.000	0.923	1.385
(iii) L_{red} / mm	1.310	0.846	1.153	1.077	1.000	0.923	1.154
$\overline{L_{red}}$ / mm	0.975	0.949	1.308	1.180	1.051	0.974	1.180
S. D.	0.396	0.0889	0.266	0.118	0.089	0.0889	0.194
**Device**	**Colored PEI with PEI**						
Ideal P / kPa	7.33	35.6	112	151	198	290	347
(i) L_{red} / mm	1.094	1.484	2.266	2.188	2.501	2.891	3.281
(ii) L_{red} / mm	1.328	1.328	2.031	2.111	2.344	2.500	4.688
(iii) L_{red} / mm	0.938	1.953	2.500	2.500	2.422	3.906	3.829
$\overline{L_{red}}$ / mm	1.120	1.588	2.265	2.266	2.422	3.099	3.933
S. D.	0.196	0.325	0.235	0.206	0.0785	0.726	0.709
**Device**	**DL containing the PEI solution**						
Ideal P / kPa	6.61	33.4	102	144	212	264	541

(i) L_{red} / mm	0.645	0.968	1.212	1.855	1.774	1.935	3.791
(ii) L_{red} / mm	0.411	0.726	2.338	1.373	1.532	2.983	3.145
(iii) L_{red} / mm	0.403	0.730	1.129	2.097	1.935	2.016	5.061
$\overline{L_{red}}$ / mm	0.486	0.808	1.560	1.775	1.747	2.311	3.999
S. D.	0.137	0.139	0.675	0.369	0.203	0.583	0.974

The color-changed depth (L_{red}) increased with an increase in P (Figure S9b and Table S9). L_{red} was measured on the cross-sectional photographs. The increment (Δx) of the reference device using c-DL/PEI was larger than that using c-DL (Figure S12c). The results indicate that the diffusion of PEI contributes to an increase in L_{red} improving the sensitivity. Moreover, the sensitivity was enhanced by the color changes of PDA (Figure S12d).

Application of unknown *P*

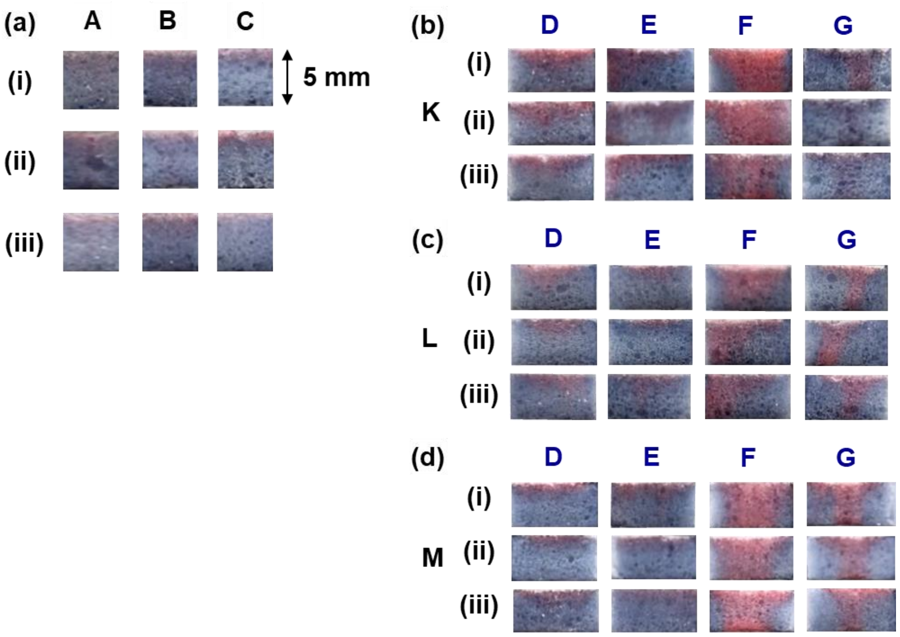

Figure S13. Cross-sectional photographs with the application of unknown *P* using an ornament of giant panda. (a) Compression with the contact of the parts A–C. (b) Compression with the contact of the parts D–G applied in the directions K–M.

Table S7. Summary of *P* and Δ*x*.

Contact	A	B	C	
(i) *P* / kPa	33.7	15.2	10.3	
(ii) *P* / kPa	38.6	15.8	11.4	
(iii) *P* / kPa	39.7	15.9	28.7	
$\bar{P}$ / kPa	37.3	15.6	16.8	
S. D.	3.19	0.379	10.3	
Contact	D	E	F	G
Press		K		
(i) *P* / kPa	188	45.9	513	46.5
(ii) *P* / kPa	174	91.8	404	16.5
(iii) *P* / kPa	123	44.3	290	16.9
$\bar{P}$ / kPa	162	60.7	402	26.6
S. D.	34.1	27.0	112	17.2
Press		L		
(i) *P* / kPa	93.5	25.1	204	122
(ii) *P* / kPa	23.7	4.18	190	145
(iii) *P* / kPa	23.4	7.51	226	153
$\bar{P}$/ kPa	46.8	12.3	207	140
S. D.	40.4	11.2	18.4	16.1
Press		M		

(i) *P* / kPa	13.1	20.7	296	170
(ii) *P* / kPa	6.98	6.65	246	105
(iii) *P* / kPa	14.9	7.95	268	188
$\bar{P}$ / kPa	11.7	11.8	270	154
S. D.	4.16	7.75	25.0	43.5

Differences in the stapled states

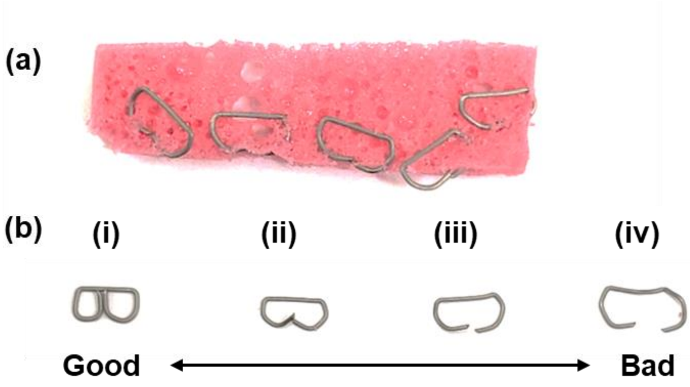

Figure S14. Photographs of the softness model III stapled with a linear stapler (a) and collected staples with the different stapled states (b).

When the softness model III was compressed and stapled using a linear stapler device, as shown in Figure 6a, the formability of the staples, *i.e.* the shape, was different (Figure S14a). Formation of B-shaped staple is required to achieve adequate stapled state ((i) in Figure S14b). On the other hand, the hard and/or thick tract causes the inadequate stapled states ((ii)–(iv) in Figure S14b). Therefore, the studies on the compression stresses applied to the tracts with the different softness and thickness are significant to avoid the inadequate stapling.

Sensing the softness models I–III

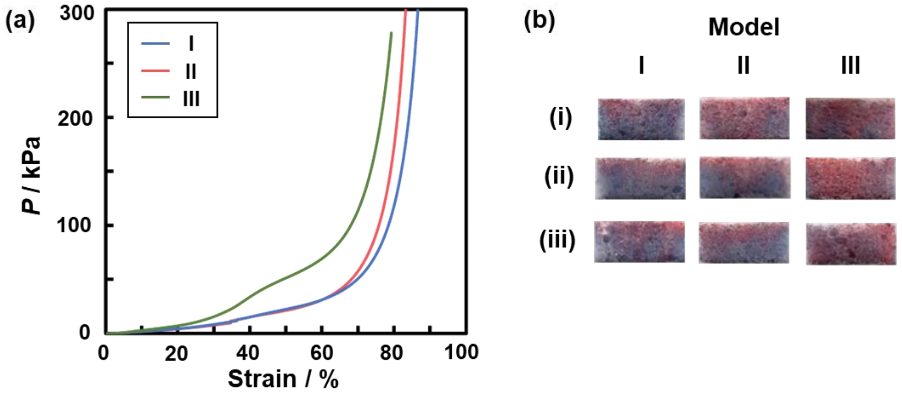

Figure S15. Softness of the models I–III and their sensing results. (a) Stress-strain curves of the PDA/DL device set between the two softness models, as shown in Figure 6b. (b) Cross-sectional photographs of the models I–III.

Table S8. Summary of P and Δx.

Softness model	I	II	III
(i) Δx / –	0.0248	0.0433	0.0449
(ii) Δx / –	0.0266	0.0345	0.0560
(iii) Δx / –	0.0286	0.0290	0.0405
$\overline{\Delta x}$ / –	0.0267	0.0356	0.0471
S. D.	0.00187	0.00720	0.00800

The stress-strain curves in Figure 6c were measured using only the softness models I–III. Figure S11a shows the stress-strain curves of the models I–III with the PDA/DL device for the sensing experiments. The melamine sponge B with dispersion of DL 25 mg was used for this experiment (Figures S7 and S8).

Stress-distribution imaging using a circular stapler

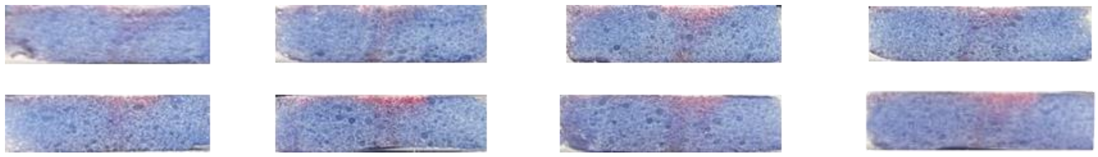

Figure S16. Cross-sectional photographs of the PDA/DL sponge device for stress-distribution imaging of the compression using automated anastomosis device with circularly arranged staples.

The profile in Figure 6k,l was prepared using the average and standard deviation based on these eight photographs.
